# *Vegetative to generative1* (*Vgt1*) is an enhancer affecting flowering time and jasmonate signaling in maize by promoting the expression of *Zea mays Related to APETALA 2.7*

**DOI:** 10.1093/plphys/kiaf468

**Published:** 2025-10-03

**Authors:** Johan Zicola, Blaise Weber, Xiaoyu Tu, Rechien Bader, Dimitrios Zisis, Stijn Aesaert, Silvio Salvi, Pawel Krajewski, Mieke Van Lijsebettens, Chuanshun Li, Yangmeihui Li, Silin Zhong, Stefan Scholten, Franziska Turck, Maike Stam

**Affiliations:** Department Plant Developmental Biology, Max Planck Institute for Plant Breeding Research, Carl-von-Linné-Weg 10, Köln 50829, Germany; Department of Crop Sciences & Center for Integrated Breeding Research (CiBreed), Georg-August-University Göttingen, Von-Siebold-Str. 8, Göttingen 37075, Germany; Swammerdam Institute for Life Sciences, University of Amsterdam, Science Park 904, Amsterdam 1098XH, The Netherlands; Shanghai Collaborative Innovation Center of Agri-Seeds, Joint Center for Single Cell Biology, School of Agriculture and Biology, Shanghai Jiao Tong University, Shanghai 200240, China; Swammerdam Institute for Life Sciences, University of Amsterdam, Science Park 904, Amsterdam 1098XH, The Netherlands; Institute of Plant Genetics, Polish Academy of Science, Strzeszyńska 34, Poznań 60-479, Poland; Department of Plant Biotechnology and Bioinformatics, Ghent University, Ghent 9052, Belgium; VIB Center for Plant Systems Biology, Technologiepark 71, Ghent 9052, Belgium; Department of Agricultural and Food Sciences, University of Bologna, Viale Fanin 44, Bologna 40127, Italy; Institute of Plant Genetics, Polish Academy of Science, Strzeszyńska 34, Poznań 60-479, Poland; Department of Plant Biotechnology and Bioinformatics, Ghent University, Ghent 9052, Belgium; VIB Center for Plant Systems Biology, Technologiepark 71, Ghent 9052, Belgium; Shanghai Collaborative Innovation Center of Agri-Seeds, Joint Center for Single Cell Biology, School of Agriculture and Biology, Shanghai Jiao Tong University, Shanghai 200240, China; Shanghai Collaborative Innovation Center of Agri-Seeds, Joint Center for Single Cell Biology, School of Agriculture and Biology, Shanghai Jiao Tong University, Shanghai 200240, China; School of Life Sciences, The Chinese University of Hong Kong, EG12 Science Centre East, Hong Kong, China; Department of Crop Sciences & Center for Integrated Breeding Research (CiBreed), Georg-August-University Göttingen, Von-Siebold-Str. 8, Göttingen 37075, Germany; Department Plant Developmental Biology, Max Planck Institute for Plant Breeding Research, Carl-von-Linné-Weg 10, Köln 50829, Germany; Swammerdam Institute for Life Sciences, University of Amsterdam, Science Park 904, Amsterdam 1098XH, The Netherlands

## Abstract

Transcriptional enhancers participate in cell and tissue differentiation in all multicellular organisms. Here, we characterized the candidate enhancer *Vegetative to generative1* (*Vgt1*), a major quantitative trait locus for flowering time in maize. Transgenic lines containing an inverted repeat that induces DNA methylation at *Vgt1* showed early flowering and an accelerated growth rate during early development. DNA methylation of *Vgt1* was associated with the downregulation of the AP2-like floral repressor *ZmRap2.7* in specific leaf tissues at the early stages of maize development. In line with *Vgt1* regulating *ZmRap2.7*, chromosome conformation capture data showed that *Vgt1* physically interacts with the *ZmRap2.7* transcription start site. Finally, chromatin immunoprecipitation of transiently expressed *ZmRap2.7* in protoplasts indicated that this transcription factor binds to the promoters of several hundred genes. These genes include many genes that are differentially expressed in maize lines with and without extra DNA methylation at *Vgt1*. Altogether, we show that *ZmRap2.7* is transcriptionally controlled by *Vgt1* and is involved in regulating flowering time and other biological pathways, such as jasmonate signaling.

## Teaser

A maize transcription factor gene is regulated by a distal enhancer and is involved in regulating flowering time and other biological pathways, such as hormone signaling and plant immunity.

## Introduction

In large genomes, such as that of *Zea mays* (maize), many cis-regulatory modules (CRMs) have moved away from their target genes by the insertion of transposable elements (TEs) (Lu et al. 2019). Importantly, genetic polymorphisms associated with plant traits are found within coding sequences as well as in CRMs, indicating the relevance of CRMs in trait adaptation (Haudry et al. 2013; Schmitz et al. 2022; Marand et al. 2023). Among CRMs, transcriptional enhancers (hereafter called enhancers) promote transcription of one or more target genes in specific cell types and developmental stages (Lu et al. 2019; Schmitz et al. 2022; Marand et al. 2023) and can be orientation dependent (Zhu et al. 2015; Voichek et al. 2024). A number of distal candidate or validated enhancers have been characterized in maize, including those associated with the *P-rr* gene (Sidorenko et al. 1999), *tassel branching1* (*tb1*) (Studer et al. 2011), the hepta-repeat controlling the anthocyanin gene *b1* (Stam et al. 2002), *KRN4* regulating *UB3* (Du et al. 2020), DICE regulating *Bx1* (Zheng et al. 2015), and a Harbinger TE regulating *ZmCCT9* (Huang et al. 2018).

Another well-studied putative distal enhancer in maize is *Vegetative to generative transition1 (Vgt1)*, a major quantitative trait locus (QTL) for flowering time in maize, mapped to a ∼2 kb region sharing conserved noncoding sequences (CNSs) with *Sorghum bicolor* (*sorghum*) and *Oryza sativa* (rice) (Vlăduţu et al. 1999; Salvi et al. 2007; Buckler et al. 2009; Song et al. 2021). *Vgt1* is located about 70 kb upstream of the *Zea mays Related to apetala2.7* (*ZmRap2.7)* gene encoding an APETALA2 (AP2)-like transcription factor (TF) that acts as a floral repressor, most likely by repressing directly or indirectly the activity of the florigen gene *ZEA CENTRORADIALIS 8 (ZCN8)* (Aukerman and Sakai 2003; Salvi et al. 2007; Liang et al. 2019). Besides *ZmRap2.7* and *ZCN8*, other genes involved in the flowering pathway include the ZCN8-interactor *delayed flowering1* (*dlf1*) and the 3 MADS-box TFs *ZmMADS4*, *ZmMADS67*, and *ZmMADS69* (Muszynski et al. 2006; Salvi et al. 2007; Danilevskaya et al. 2008; Meng et al. 2011; Minow et al. 2018; Liang et al. 2019).

Sequence variations at *Vgt1* were associated with early flowering and downregulation of *ZmRap2.7*, suggesting *Vgt1* contains enhancer activity regulating *ZmRap2.7* expression (Salvi et al. 2007; Ducrocq et al. 2008; Castelletti et al. 2014). *Vgt1* displays several enhancer hallmarks such as chromatin accessibility (Rodgers-Melnick et al. 2016; Ricci et al. 2019; Tu et al. 2020), expression of a long noncoding RNA (Li et al. 2014; Schmitz et al. 2022), and the binding of multiple TFs (Tu et al. 2020). Furthermore, Hi-C and HiChIP (Hi-C combined with chromatin immunoprecipitation [ChIP] (Mumbach et al. 2016)) experiments showed that *Vgt1* and *ZmRap2.7* physically interact (Li et al. 2019a; Ricci et al. 2019; Xu et al. 2020).

We here demonstrate that *Vgt1* regulates *ZmRap2.7* expression as a *bona fide* enhancer. We also show that *ZmRap2.7* does not only affect flowering time but also directly regulates the expression of several hundreds of genes involved in other pathways such as jasmonate signaling and plant immunity.

## Results

### Epigenetic inactivation of *Vgt1* is associated with early flowering and accelerated growth

**Figure 1.**
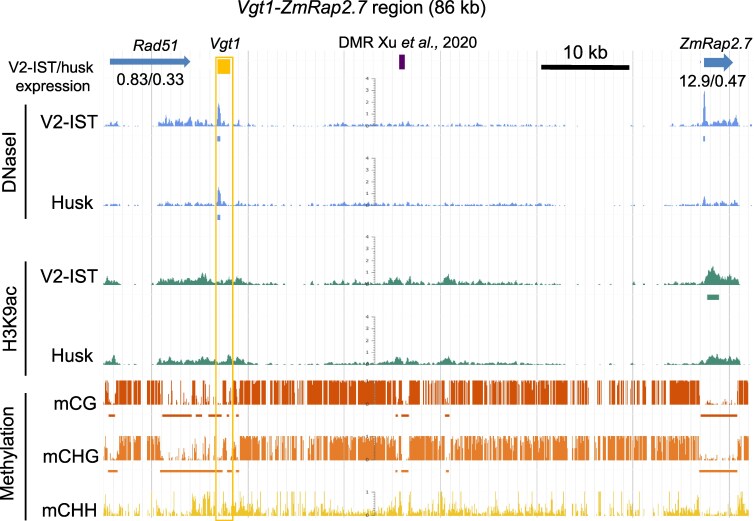
DNA and chromatin characteristics of *Vgt1* and *ZmRap2.7* regions in B73 growth stage 2 inner stem (V2-IST) and husk tissue. From top to bottom: regions of interest (B73 AGPv4 coordinates, chr8:135,927,630..136,013,943) with *Vgt1, DMR* and B73 AGPv4 genes (Rad51 and ZmRap2.7), normalized gene expression levels in V2-IST and husk (reads per thousand million mapped reads [RPKM], most expressed isoform), DNaseI hypersensitivity (DNaseI) and H3K9ac enrichment (horizontal bars indicate significant coverage), DNA methylation levels in CG (red), CHG (H = C, A, or T) (orange), and CHH context (yellow) (Oka et al. 2017). DNA methylation data are from coleoptile tissue of 5-d-old seedlings. For DNase I hypersensitivity and H3K9ac enrichment, the coverage shown is based on 2 and 3 replicates pooled per tissue, respectively. UMRs are indicated by bars below the DNA methylation levels. The vertical yellow box indicates the *Vgt1* coordinates.

In previous studies, we identified genome-wide enhancer candidates in maize (accession B73) based on parameters that these regions exhibited, i.e. a simultaneous presence of accessible chromatin and H3K9ac, in combination with the absence of DNA methylation (Oka et al. 2017, 2020). However, the list did not include *Vgt1* because it did not overlap with H3K9ac (Fig. 1A). Our data on DNase I hypersensitive sites (DHS) and unmethylated regions (UMRs) (Oka et al. 2017) did show that *Vgt1* overlapped with a DHS in the 2 tissues examined, namely, the inner stem tissue of V2 seedlings (V2-IST) and husk leaves. Additionally, *Vgt1* contained 2 UMRs, one of which overlapped with the DHS site (Fig. 1).

**Figure 2.**
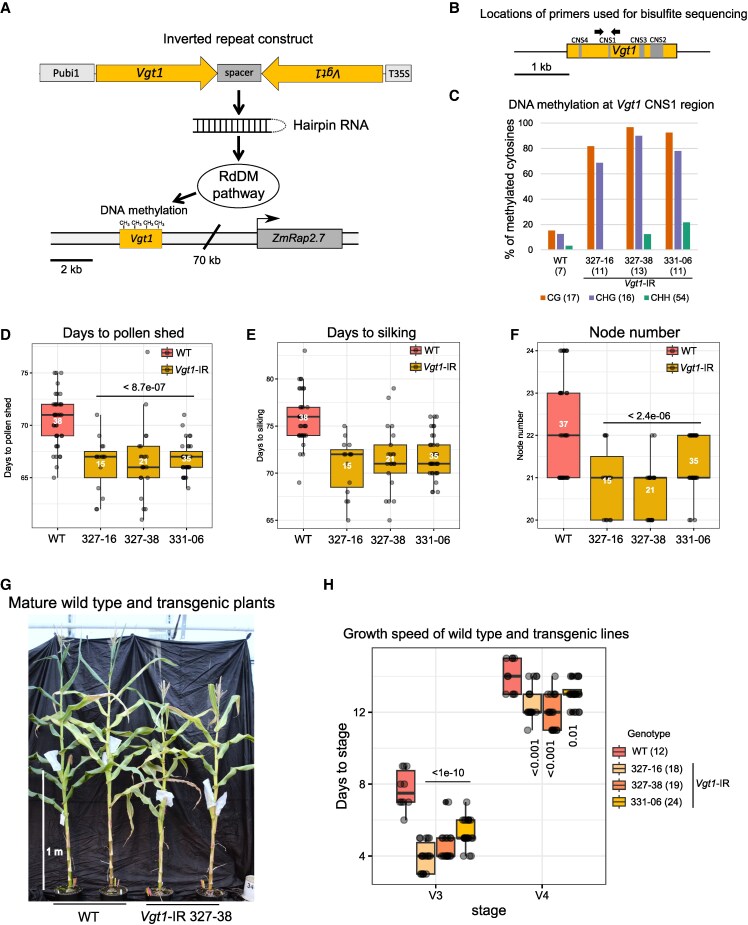
*Vgt1*-IR construct triggers early flowering and enhanced growth speed. **A)** Schematic representations of IR construct used to generate *Vgt1*-IR transgenic lines. Pubi1 refers to the promoter (P) of the maize *ubiquitin 1* gene (GRMZM2G409726) and T35S to the terminator (T) sequence of the Cauliflower Mosaic Virus 35S RNA gene (construct details in Supplementary Fig. S1). **B)** Schematic representation of *Vgt1* indicating the primers (black arrows) used for PCR amplification of the region analyzed by bisulfite sequencing. Note that no distinction could be made between the endogenous and transgenic *Vgt1* sequence. Gray areas within *Vgt1* indicate CNSs defined in Salvi et al. (2007). **C)** CG, CHG, and CHH DNA methylation levels at CNS1 of *Vgt1* in WT and *Vgt1*-IR transgenic lines. The numbers in parentheses below each maize line and besides the methylation context indicate the number of clones sequenced (*n* ≥ 7) and the number of cytosines in each context in the examined amplicon, respectively. Percent of methylation is defined by the ratio of methylated cytosines over total number of cytosines in a specific context in the sequenced clones. Flowering time in DPS **D)**, days to silking **E)**, and node number **F)** for WT and 3 *Vgt1*-IR transgenic lines. *P*-values and number of plants genotyped are indicated in the plots (*n* ≥ 15). **G)** Representative mature maize plants for WT and *Vgt1*-IR line 327-38. The white vertical bar indicates the scale. **H)** Growth speed of WT and 3 *Vgt1*-IR lines. The box plots show the number of days each line took to reach growth stages V3 and V4 taking the moment of reaching V2 as a start. Number of plants per independent transgenic line is indicated in the legend between parentheses (*n* ≥ 18). For the plots in **D** to **F)** and **H)**, ANOVA followed by a Dunnett's test (2-sided) was performed to compare each genotype to WT. Center lines of box plots show the medians, box limits the 25th and 75th percentiles, whiskers extend 1.5 times the interquartile range from the 25th and 75th percentiles, and dots individual data points.

Besides SNPs and indels, early flowering was associated with insertion of a miniature inverted-repeat TE (MITE) into *Vgt1* (Salvi et al. 2007). Sequences flanking the MITE showed increased DNA methylation levels compared to a line lacking the MITE (Castelletti et al. 2014). Early flowering could be due to the MITE insertion itself, e.g. disrupting TF binding sites, or to the DNA methylation changes observed. To assess whether DNA methylation at *Vgt1* affects *ZmRap2.7* expression, we generated transgenic lines carrying an inverted repeat (IR) targeting *Vgt1* (Fig. 2A; Supplementary Fig. S1) in the B104 inbred line, a line lacking the MITE (Coussens et al. (2012). We used the maize inbred B104 because it derives from the same populations than B73 (93% similarity) and is, in contrast to B73, a transformable maize line standardly used in the maize community (Manchanda et al. 2021; Kang et al. 2022). Importantly, the sequence of *Vgt1* is identical, and that of the entire 69.7 kb *ZmRap2.7* locus is nearly identical in the 2 maize lines (see Materials and methods section). Three independent *Vgt1*-IR transgenic maize lines, 327-16, 327-38, and 331–06, each segregating for one *Vgt1*-IR locus, were retained for further analyses. Expression of IR constructs can mediate the epigenetic inactivation of promoters and/or CRMs through RNA-directed DNA methylation (RdDM) (Mette et al. 2000), providing a tool to study the role of regulatory sequences of interest (Sidorenko et al. 2009; Arteaga-Vazquez et al. 2010; Zicola et al. 2019).

We then assessed the DNA methylation level at *Vgt1* for the 3 independent transgenic lines shown in Fig. 2 and non-transgenic B104 (WT), using targeted bisulfite sequencing on genomic DNA from leaf tissue. For the *Vgt1*-IR lines, the methylation status at the CONSERVED NON-CODING SEQUENCE 1 (CNS1) region was investigated, as this particular CNS is disrupted by the insertion of a MITE in early flowering lines (Salvi et al. 2007). This analysis revealed an increased DNA methylation level in CG and CHG context in all 3 *Vgt1*-IR lines compared to WT (Fig. 2, B and C). Note that methylation was not assessed over the whole *Vgt1* IR target region, but previous work indicates that induced DNA methylation is specifically restricted to the IR target region and that spreading is minimal (Přibylová et al. 2019; Zicola et al. 2019).

Knowing *Vgt1* was epigenetically modified, we examined flowering time of the IR transgenic lines by measuring days to pollen shed (DPS), days to silking, and node number and found a clear early phenotype for all 3 *Vgt1-*IR lines tested (Fig. 2, D to G; Supplementary Fig. S2, A to C). The early flowering phenotypes of *Vgt1*-IR lines could either be the consequence of the reduced number of nodes in *Vgt1*-IR lines compared to non-transgenic wild type B104 (WT), a faster growth, or a combination of the 2. Therefore, we examined the effect of *Vgt1* methylation on developmental timing by counting the number of days the plants required to reach the different developmental vegetative (V) stages (Abendroth et al. 2011), starting from stage V2 (Fig. 2H; Supplementary Fig. S3A). As comparison, we included C22-4 and N28 as used previously (Salvi et al. 2007), which appeared the fastest and slowest growing and flowering lines, respectively (Supplementary Fig. S3, A to E). Interestingly, *Vgt1*-IR lines were characterized by a significant growth acceleration from stage V2 to V3, reaching V3 3 to 4 d earlier than WT (Fig. 2H; Supplementary Fig. S3B). After stage V4, developmental timing in most *Vgt1*-IR lines became more similar to that in WT B104 so that differences between the lines became less significant with time (Supplementary Fig. S3A).

In conclusion, DNA methylation of *Vgt1* results in early flowering and faster growth, in line with *Vgt1* regulating ZmRap2.7 expression.

### Epigenetic inactivation of *Vgt1* leads to downregulation of *ZmRap2.7* expression

**Figure 3.**
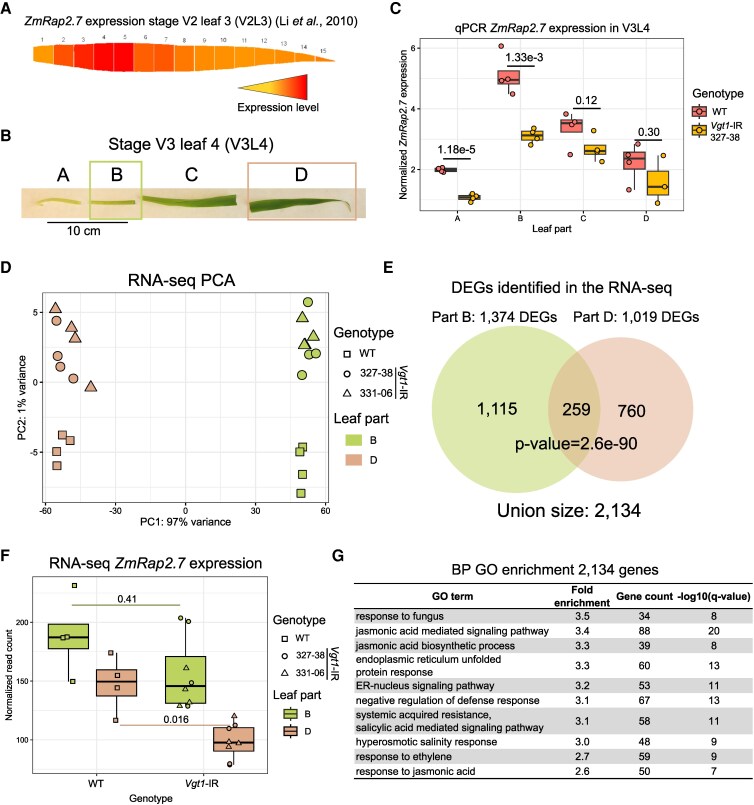
*ZmRap2.7* expression levels are downregulated in *Vgt1*-IR lines. **A)** Expression levels of *ZmRap2.7* across 15 sections of leaf 3, stage 2 plants (V2L3) based on published data from (Li et al. 2010) and observed on the maize eFP browser (Hoopes et al. 2019). **B)** Maize WT leaf 4, stage V3, showing the 4 separate leaf parts analyzed by RT-qPCR. The leaf collar is at the cut site between part B and C. Parts B and D were used for RNA-seq. **C)** Expression of *ZmRap2.7* measured by RT-qPCR in the 4 V3L4 tissue parts of WT and 1 *Vgt1*- IR transgenic line, with *P*-values comparing WT and transgenic plants indicated on the plot (*t*-test, *n* = 4 biological replicates). **D)** PCA of RNA-seq data for the different genotypes and leaf parts (*n* = 4 biological replicates). **E)** Venn diagram of the intersect of DEGs in leaf parts B and D with the *P*-value indicating the significance of the intersect (Fisher's exact test). **F)** Normalized expression levels of *ZmRap2.7* between WT and IR lines. Adjusted *P*-values are derived from the Wald test (DESeq2 R package) and indicated on the boxplot. **G)** GO enrichment analysis for biological processes performed for the 2,134 DEGs identified between *Vgt1*-IR and WT. Only the top 10 GO terms with the lowest *q*-values are displayed, sorted by decreasing fold enrichment value. Full GO term enrichment results in Supplementary Table S2. Center lines of box plots show the medians, box limits the 25th and 75th percentiles, whiskers extend 1.5 times the interquartile range from the 25th and 75th percentiles, and small circles, squares, and triangles represent individual data points.

To test if *Vgt1* methylation resulted in downregulation of the presumed target gene *ZmRap2.*7 (Salvi et al. 2007), we investigated which tissues and developmental stages should be examined. We first determined the timing of the vegetative to reproductive meristem transition in B104 and found it takes place between stage V6 and V7 (Supplementary Fig. S4A). Furthermore, in line with published data (Takacs et al. 2012), in situ hybridization with a *ZmRap2.7* probe on shoot apices of V3 plants indicated higher *ZmRap2.7* expression in leaf primordia and leaf tissue compared to the meristem (Supplementary Fig. S4B). We then analyzed published transcriptomic data of 15 sections of the 3rd leaf of 9-d-old (V2 stage) B73 seedlings (Li et al. 2010) and observed that *ZmRap2.7* expression follows a gradient across the leaf with the highest expression in sections 3–5 of the leaf and a lower expression toward the tip (Fig. 3A). In addition, 2 other flowering time players, *ZCN8* and *ZmMADS4*, were shown to be more expressed at the leaf tip and base, respectively (Danilevskaya et al. 2008; Meng et al. 2011).

Based on these observations, we measured the expression dynamics of *ZmRap2.7*, *ZCN8*, and *ZmMADS4* across 4 leaf parts of the next emerging leaf at 3 developmental stages (V3 to V5) of WT and the *Vgt1*-IR line 327-38 using RT-qPCR (Fig. 3B; Supplementary Fig. S5, A to C). *ZmRap2.7* expression was highest and most different between non-transgenic B104 and line 327-38 in leaf part B of stage V3 leaf 4 (V3L4) (Fig. 3C; Supplementary Fig. S5A). *ZmRap2.7* expression was significantly downregulated in the *Vgt1*-IR line compared to WT in this leaf part. *ZCN8* was, even at this pre-transition stage, upregulated in the transgenic plants in the distal leaf part at all stages, with significant differences at stages V3 and V4 (Supplementary Fig. S5B). An effect on the expression of *ZmMADS4*, a more downstream gene in the flowering pathway, became only more apparent in later development stages (Supplementary Fig. S5C). These data indicate that all 3 flowering players examined show trends of differential expression in *Vgt1*-IR line 327-38, with *ZmRap2.7* being downregulated and *ZCN8* and *ZmMADS4* being upregulated, in accordance with the early flowering phenotype observed in the transgenic plants.

To identify other potential genes affected by *Vgt1* inactivation, we performed RNA-seq on 2 V3L4 leaf parts (B and D) (Fig. 3B) of 2 *Vgt1*-IR transgenic lines (327-38 and 331-06) and WT, with 4 biological replicates each. When examining the overall variability of gene expression using principal component analysis (PCA), most variance (97%) was explained by PC1 associated with the leaf part (Fig. 3D), while PC2 associated with the genotype explained 1% of the variance. Since the transgenic lines clustered together in the PCA, and apart from the WT control, we considered them as biological replicates. Indeed, we found 1,374 and 1,019 significant differentially expressed genes (DEGs) in leaf part B and D between WT and *Vgt1*-IR lines, respectively, and a total of 2,134 DEGs across the 2 leaf parts (FDR < 5%, no expression fold-change threshold) (Fig. 3E; Supplementary Table S1). *ZmRap2.7* was among the DEGs in leaf part D, where it is significantly downregulated in the *Vgt1-IR* transgenic lines (Fig. 3F). Although downregulation also seems to happen in leaf part B, the difference is nonsignificant due to high variance between replicates (Fig. 3F). *ZCN8* was not significantly differentially expressed, but the pattern of expression followed the RT-qPCR results, with *ZCN8* being more expressed in leaf part D for the *Vgt1*-IR lines (Supplementary Fig. S6). We performed a gene ontology (GO) enrichment analysis with the DEGs identified across the 2 leaf parts (*n* = 2,134) and found significant signals for GO terms related to jasmonic acid, ethylene, salicylic acid, defense response, and hyperosmotic stresses (Fig. 3G; Supplementary Table S2).

Altogether, our results indicate that the IR targeting *Vgt1* negatively affects *ZmRap2.7* expression and that *ZmRap2.7* regulates the expression of other flowering time genes such as *ZmMADS4* and *ZCN8*, but also thousands of other genes. The latter are enriched for genes related to stress response and hormonal signaling, especially jasmonate.

### ZmRap2.7 ChIP-seq analysis

**Figure 4.**
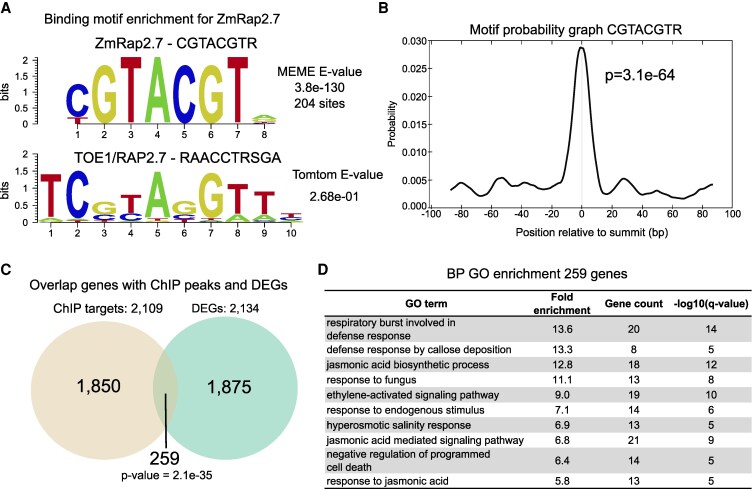
ChIP-seq analysis of ZmRap2.7 binding sites. **A)** Top enriched binding motif based on MEME-ChIP analysis compared to the TARGET OF EAT 1 (TOE1) binding site found in Arabidopsis (Franco-Zorrilla et al. 2014). *E*-values are indicated in the plot. The *y* axis represents information content in bits. **B)** Central enrichment of the top enriched motif based on Centrimo analysis for the MEME motif. *E*-value is indicated in the plot. **C)** Venn diagram of the overlap between the 2,109 genic regions containing ZmRap2.7 peaks from 10 kb upstream of the TSS to 5 kb downstream of the TTS (ChIP targets) and the 2,134 DEGs identified between WT and *Vgt1*- IR lines (Fig. 3E). The significance of the overlap was tested with a Fisher's exact test (one-sided). **D)** GO enrichment analysis for biological processes performed for the 259 genes containing ChIP-seq peaks between 10 kb upstream of the TSS to 5 kb downstream of the TTS and that are differentially expressed between WT and *Vgt1*-IR lines. Only the top 10 GO terms with the lowest *q*-values are displayed, sorted by decreasing fold enrichment value. Full GO term enrichment results in Supplementary Table S5.

Since we found 2,134 DEGs in the *Vgt1*-IR transgenic lines, it was assessed whether these genes could be directly regulated by *ZmRap2.7* using ChIP-seq on B73 maize leaf protoplasts transiently expressing a tagged recombined ZmRap2.7 protein. About 2,200 reproducible peaks were identified across 2 biological replicates and the top 1,000 were used to identify enriched DNA motifs (Supplementary Table S3). We found the centrally enriched CGTACGTR motif in 20% (204/1,000) of the peaks (Fig. 4A). This motif is relatively close to that of the Arabidopsis (*Arabidopsis thaliana*) ortholog TOE1/RAP2.7 (Fig. 4A) and was centrally enriched within the peaks (Fig. 4B). The second most abundant motif was found in 13.6% of the peaks and matched the CGCCGCCG found in another AP2/ERF Arabidopsis TF, DREB AND EAR MOTIF PROTEIN 3 (DEAR3) (Supplementary Fig. S7A). This motif is also matching the most significant binding motifs of 14 maize ETHYLENE-RESPONSIVE ELEMENT BINDING (EREB) TFs reanalyzed from published ChIP-seq data (Tu et al. 2020) and also from the AP2/ERF TF family (Franco-Zorrilla et al. 2014) (Supplementary Fig. S7B). In conclusion, ZmRap2.7 can associate with several motifs, including a related motif bound by the Arabidopsis ortholog TOE1/RAP2.7, and a motif bound by Arabidopsis and maize EREB TFs.

We next looked at the regions bound by ZmRap2.7 and found that it binds preferentially around transcriptional start sites (TSS) (Supplementary Fig. S7C). We identified 2,109 genes containing peaks within 10 kb upstream of the TSS to 5 kb downstream of the transcription termination site (Supplementary Table S4). GO term analyses for the 2,109 genic regions containing ZmRap2.7 peaks revealed enrichment of similar GO terms than for the 2,134 DEGs in the *Vgt1-*IR lines (Supplementary Fig. S8A; Fig. 3G; Supplementary Table S5), supporting the idea that ZmRap2.7 is regulating genes in these pathways. In addition, 259 of ZmRap2.7-bound genes were also differentially expressed in the *Vgt1-*IR lines, supporting that *Vgt1* inactivation affects genes that are bound and regulated by *ZmRap2.7* (Fig. 4C). A GO term enrichment analysis for the 259 overlapping genes mostly found terms related to biotic and abiotic stress responses including ethylene and jasmonate signaling and jasmonate biosynthesis (Fig. 4D).

In Arabidopsis, TOE1/RAP2.7 regulates flowering by binding at the promoter of the ortholog of *ZCN8*, *FLOWERING LOCUS T* (*FT*). Therefore, we looked whether ZmRap2.7 binds near *ZCN8* and found a binding peak at an open chromatin region 11 kb upstream, with 2 genes in between (Supplementary Fig. S8, B and C). The motif within the peak is the TOE1/RAP2.7-like CGTACGTT motif (Supplementary Fig. S8B). This peak may indicate a CRM that enables ZmRap2.7 to regulate *ZCN8*. The 2 genes in between are not differentially expressed in the *Vgt1-IR* lines.

### *Vgt1* physically interacts with its target gene *ZmRap2.7*

Since enhancer elements are known to modulate gene expression through physical interactions with the promoter of their target gene, we looked at potential interactions between *ZmRap2.7* and *Vgt1* by Circular Chromosome Conformation Capture sequencing (4C-seq) (Simonis et al. 2006; Zhao et al. 2006), using *Bgl*II as first, and *Csp*6I as second restriction enzyme. To define whether potential contacts are associated with *ZmRap2.7* expression levels, we used 2 tissues, inner stem tissue from V2 seedling (V2-IST) and husk tissue, which exhibit high (12.92 reads per kilobase of transcript per million mapped [RPKM]) and low (0.47 RPKM) *ZmRap2.7* expression levels, respectively (Oka et al. 2017). We generated 4C-seq libraries for the 2 tissue types using the transcription start site (TSS) region of *ZmRap2.7* as a viewpoint. We define interacting peaks as *Bgl*II restriction sites preferentially ligating with the *Bgl*II restriction site of the viewpoint.

**Figure 5.**
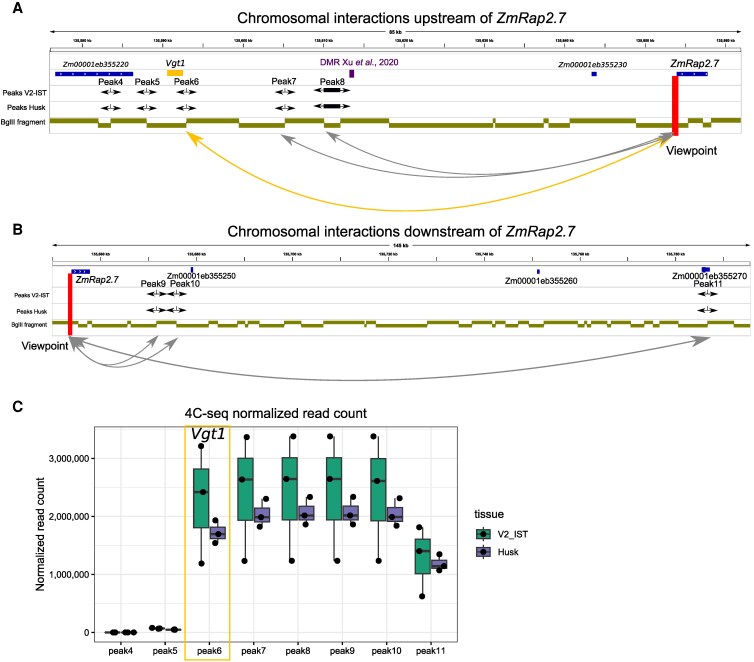
Chromosomal contacts involving the *ZmRap2.7* TSS in B73. Chromosomal interactions upstream **A)** and downstream **B)** of *ZmRap2.7* in stage 2 inner stem tissue (V2-IST) and husk tissue using the TSS region of *ZmRap2.7* as viewpoint (chr8:135,653,433-135,654,281, vertical, thick red line). Black horizontal arrows indicate the read mapping direction on either end of *Bgl*II restriction fragments showing significant interaction levels (called peaks). The black box between 2 black arrows represents a fragment lacking a *Csp6*I restriction site (i.e. blind fragment). Fourteen and 12 peaks were found in V2-IST and husk, respectively, of which 12 are common and 2 unique to V2-IST (peak 1 and 14). Peaks 4 to 11 are shown here; all peaks are shown in Supplementary Fig. S9. Khaki boxes represent *Bgl*II restriction fragments. The purple box indicates the DMR region found to interact with *Vgt1* in published Hi-C data (Li et al. 2019a). **C)** Normalized read count for significant peaks 4 to 11 in both tissues. Center lines of box plots show the medians, box limits the 25th and 75th percentiles, whiskers extend 1.5 times the interquartile range from the 25th and 75th percentiles, and dots represent individual biological replicates (*n* = 3).

We identified 12 significant peaks in both tissues and 2 additional peaks unique to V2-IST (Fig. 5, A and B; Supplementary Fig. S9A and Table S6) within a 486 kb region surrounding *ZmRap2.7*. Peak 6 is 400 bp downstream of *Vgt1*, at the 3′ end of a *Bgl*II fragment overlapping *Vgt*I (Fig. 5A). We also found a peak at the 5′ end of a differentially methylated region (DMR) reported to interact with *Vgt1* in 2-wk-old B73 leaves based on Hi-C data (Li et al. 2019a) (peak 8) (Fig. 5A). In addition, the *ZmRap2.7* TSS interacts with a gene annotated as a ribosomal protein (Zm00001eb355270, ribosomal protein L10/acidic P0) located 128 kb downstream (peak 11) (Fig. 5B). Together, our results support the interaction of *Vgt1* with the *ZmRap2.7* TSS and reveals additional interacting regions upstream and downstream of *ZmRap2.7*.

When comparing the strength of the interactions between both tissues, we found higher interaction levels, although not significant, for V2-IST compared to husk (Fig. 5C), in line with the higher expression of *ZmRap2.7* in V2-IST. Interestingly, when examining all 14 peaks, the peaks from peak 6, downstream of *Vgt1*, up to peak 11 (Zm00001eb355270), 200 kb downstream of *ZmRap2.7*, show high normalized read counts, while the peaks flanking this region show much lower normalized read counts (Fig. 5C; Supplementary Fig. S9, A and B), suggesting that both *Vgt1* and Zm00001eb355270 are located at the borders of relatively frequent chromatin loops that include the *ZmRap2.*7 TSS.

Altogether, our results indicate several strong interactions between the *ZmRap2.7* TSS and upstream and downstream regions, including *Vgt1*, indicating enhancer–promoter interactions between *Vgt1* and the *ZmRap2.7* core promoter. This interaction was found in both tissues, suggesting *Vgt1* may constitutively interact with the *ZmRap2.7* core promoter, being active in V2-IST but repressed in husk. In addition, *Vgt1* is located at the 5′ border of a genomic region showing strong interactions with *ZmRap2.7*.

## Discussion

Although several groups have attempted genome-wide identification of candidate enhancers using NGS (Oka et al. 2017; Ricci et al. 2019), validating enhancer function remains challenging, especially in difficult to transform species. In this study, we show that the candidate enhancer region *Vgt1* indeed acts as a transcriptional enhancer of the floral repressor gene *ZmRap2.7*. We functionally analyzed *Vgt1* through its inactivation by RdDM induced by a *Vgt1*-IR transgene. In *Vgt1-IR* transgenic maize lines, the expression of the *ZmRap2.7* flowering time repressor was significantly reduced, which was associated with early flowering and accelerated growth. Furthermore, 4C-seq showed chromosomal interactions between *Vgt1* and the core promoter of *ZmRap2.7*. This study also shows that *Vgt1* inactivation affects the expression of thousands of genes, many of which play a role in processes involving stress-related hormones such as ethylene, salicylic acid, and, most significantly, jasmonate. Our ChIP-seq data showed that a significant portion of these DEGs are bound by ZmRap2.7, indicating they are directly regulated by ZmRap2.7.

*Vgt1*-IR transgenic lines flowered earlier than WT plants, in accordance with the previously observed early flowering in *ZmRap2.7* knockdown and knockout plants (Salvi et al. 2007; Liang et al. 2019). The 2- to 3-d difference in flowering observed in the transgenic plants was comparable to the effect of natural variation associated with the presence of a MITE element, which is proposed to disrupt *Vgt1* function (Salvi et al. 2007; Buckler et al. 2009). The MITE insertion was associated with DNA methylation deposition at *Vgt1*, suggesting the insertion may affect TF binding via DNA methylation or sequence disruption (Castelletti et al. 2014). Our observation that induced DNA methylation affects *Vgt1* function indicates that the MITE may also affect the enhancer function via DNA methylation. We show that DNA methylation of *Vgt1* is associated with downregulation of *ZmRap2.7* and upregulation of *ZCN8* and *ZmMADS4*, supporting a model where the flowering repressor *ZmRap2.7* directly and/or indirectly negatively regulates the expression of *ZCN8* and *ZmMADS4*, 2 genes acting downstream of *ZmRap2.7* (Meng et al. 2011; Dong et al. 2012; Liang et al. 2019).

In line with enhancer function, chromosomal interactions of *Vgt1* with *ZmRap2.7* were previously demonstrated by Hi-C and HiChIP experiments (Li et al. 2019a; Ricci et al. 2019; Xu et al. 2020). Using 4C-seq, with *ZmRap2.7* TSS as viewpoint, we indeed observed relatively strong chromosomal interactions with *Vgt1* in inner stem tissue of young seedlings, in which *ZmRap2.7* is relatively highly expressed, but also in husk tissue, in which there are low *ZmRap2.7* transcript levels. There are multiple potential explanations for the persistent chromosomal interactions in husk. Firstly, the interactions found in husk may be related to Polycomb-dependent transcriptional repression. Transcription repression through Polycomb repressive complexes, which is intimately associated with H3K27me3, is shown to be associated with chromosomal interactions (Liu et al. 2016; Huang et al. 2021; Sun et al. 2023). In agreement, Ricci et al. (2019) observed high H3K27me3 levels at the *ZmRap2.7 locus*, including *Vgt1*, in ear tissue, which is formed alongside husk tissue (Supplementary Fig. S10). Therefore, we may have similar chromosomal interactions in both tissues while having 2 different expression states, active in seedling and inactive in husk. Secondly, *ZmRap2.7* transcripts may be produced in both tissues, but in husk *ZmRap2.7*, mRNA is degraded through microRNA-mediated decay. MicroRNA miR172 is expected to target *ZmRap2.7* transcripts via the ageing pathway (Dong et al. 2012). The Arabidopsis and tobacco *ZmRap2.7* ortholog is regulated by *miR172* (Aukerman and Sakai 2003; Yin et al. 2019). MicroRNA172 is present in maize, and the miR172 binding site is present in the *ZmRap2.7* transcript, making it likely to also be regulated by miR172 in maize (Salvi et al. 2007). This could explain why low *ZmRap2.7* expression in husk tissue is still associated with chromosomal interactions at the locus.

Epigenetic inactivation of *Vgt1* resulted in partial downregulation of *ZmRap2.7* expression, not in complete silencing. The *ZmRap2.7* gene is indeed not only regulated by *Vgt1*. When comparing DNA methylation levels between maize (B73) and teosinte, Xu et al. (2020) found a DMR, *Vgt1*-DMR, 20 kb downstream of *Vgt1* and 40 kb upstream of the *ZmRap2.7* TSS, that is unmethylated in B73. In line with enhancer activity, *Vgt1*-DMR enhanced reporter gene expression in a transient assay, co-localizes with H3K27ac enrichment in protoplasts and shoot tissue, and is indicated to physically interact with *Vgt1* (Li et al. 2019a; Tu et al. 2020; Xu et al. 2020). Our 4C-seq experiments showed that *ZmRap2*.*7* not only interacts with *Vgt1*, but also with the *Vgt1*-DMR region, in line with a model in which both elements play a role in regulating *ZmRap2.7* expression. Recent data also indicate there may even be more regulatory elements at *ZmRap2.7* (Engelhorn et al. 2023). Targeting these other (candidate) CRMs with DNA methylation or CRISPR-Cas-induced deletions, separate or together with *Vgt1*, can indicate their individual and combined contributions to regulating *ZmRap2.7* expression.

Using ChIP-seq to identify ZmRap2.7 binding sites across the genome, we found that the most common binding motif was relatively similar to RAP2.7/TOE1, the closest ZmRap2.7 paralog identified in Arabidopsis (Salvi et al. 2007). In addition, we found a motif close to the main binding motif shared across 14 different EREB TFs (Tu et al. 2020), indicating that ZmRap2.7 can also associate with motifs recognized by other AP2/ERF TFs, potentially increasing the regulatory possibilities of this TF family.

In Arabidopsis, RAP2.7/TOE1 regulates flowering by repressing *FT* expression. Accordingly, ZmRap2.7 binds 11 kb upstream of the *FT* homolog in maize, *ZCN8*. The binding site and *ZCN8* are, however, separated by 2 other genes. This is not uncommon and has been observed more often in eukaryotes, including maize (Ghavi-Helm et al. 2014; Li et al. 2019a). For example, in maize, the putative maize enhancer *Distal Cis-Element* (*DICE*) is located 140 kb and one gene away from its presumed target gene (Zheng et al. 2015). The occurrence of an interaction between the ZmRap2.7 binding motif and *ZCN8* could be tested by using 4C-seq with the *ZCN8* TSS as a bait or by deleting the binding motif with CRISPR-Cas9 and measure *ZCN8* expression. Note that the effect of ZmRap2.7 on *ZCN8* expression could also be indirect, for example, via another TF that is directly controlled by ZmRap2.7.

AP2/ERF TFs are shown to be key regulators of growth and development and play a crucial role in biotic and abiotic stress responses (reviewed in Xie et al. (2019) and Ma et al. (2024)). The DEGs in *Vgt1-IR* transgenic plants, genes associated with binding peaks of ZmRap2.7, and the overlap between both indicate that ZmRap2.7 plays a role in multiple biological processes. We observed a strong enrichment for pathways related to jasmonate and ethylene signaling, jasmonate biosynthesis, immunity, and hyperosmotic salinity response. In this study, we indicate a role for ZmRap2.7 in directly regulating genes in these pathways. Jasmonate and ethylene have been shown to repress flowering (Beydler et al. 2016; Kazan and Lyons 2016; Zhao et al. 2022). This report shows that an AP2/ERF TF directly regulates jasmonate biosynthesis and jasmonate and ethylene signaling genes. AP2/ERF TFs have, however, been indicated to regulate plant disease resistance genes (Ma et al. 2024). For example, Arabidopsis *ERF9* is indicated to bind the promoter of the *PATHOGEN INDUCIBLE PLANT DEFENSE* gene *PDF1.2* (Maruyama et al. 2013). In addition, ZmRap2.7 may, like other AP2/ERFs, regulate plant disease resistance through hormonal signaling (Ma et al. 2024). AP2/ERF TFs are also shown to be involved in response to salt stress (Xie et al. 2019). For example, overexpression of the AP2/ERF maize TF ZmEREB20 (Zm00001eb195920) and Lotus *LcERF056* increase salt tolerance in Arabidopsis (Fu et al. 2021; Wang et al. 2021). *LcERF056* was in addition shown to bind cis-regulatory elements of reactive oxygen species-related genes that are closely related to salt stress. Besides the pathways we observed, *ZmRap2.7* has been implicated in brace root development, with a decrease in the number of brace roots observed in a Mu insertion line (W22 background) (Li et al. 2019b).

In conclusion, our study demonstrates that *Vgt1* displays genuine enhancer activity, regulating *ZmRap2.7* and thereby flowering time, and a range of other interconnected pathways. Our study provides a foundation for future research into the role of ZmRap2.7 in biotic and abiotic stress responses. Future investigation of the genes indicated to be directly regulated by ZmRap2.7 will further elucidate the gene regulatory networks underlying tolerance to environmental stresses.

## Materials and methods

### Plant material and growth conditions

Maize plants were grown in the greenhouse at 2 different locations: the Max Planck Institute for Plant Breeding Research in Cologne (MPIPZ) and the University of Amsterdam (UvA). At the MPIPZ, maize plants were grown for flowering time experiments. At the UvA, maize plants were grown for 4C, RNA expression analysis, and flowering time and growth speed experiments. At both locations, plants were grown in soil with a 16 h light/8 h dark lighting schedule, at temperatures ranging from 20 to 25 °C and 50% to 60% humidity.

4C experiments were performed in maize line B73, in V2-IST, and husk leaves tissues. RT-qPCR experiments were performed in leaf tissues from the V2 up to the V5 stage from B104, N28, and C22-4 maize lines as well as the indicated IR lines (B104 background). Flowering time experiments were performed using B104, N28, C22-4, and transgenic IR lines. Growth rate experiments were performed on non-transgenic B104 as well as T2 progeny from *Vgt1*-IR lines, as well as the maize lines N28 and C22-4. RT-qPCR experiments were performed on T4 progeny from *Vgt1*-IR lines. Bisulfite experiments were performed on the T1 progeny (T0 transgenic × B104) of IR lines, apart from the *Vgt1-*IR line 327-38 (seeds from T4 homozygous plants).

### Inverted-repeat transgenic lines

We generated maize transgenic lines containing an IR construct targeting Vgt1 (Supplementary Fig. S1, A and B) in the inbred line B104 (Maize GDB accession no PI594047 (Hallauer et al. 1997)). We first compared the sequence of the *Vgt1-ZmRap2.7* locus (69.7 kb) from B73 (chr8:134,881,000-134,950,000 in B73 NAM5 coordinates) with that of B104 by blasting it to the B104 genome from Corteva (version 1.0, GenBank assembly GCA_910593975) using blastn (v2.11.0) (Supplementary Table S7). The *Vgt1-ZmRap2.7* locus appeared identical apart from a 2 bp deletion (chr8:135,614,910 in B73 NAM5 coordinates) and a 1 bp insertion (chr8:135,635,997 in B73 NAM5 coordinates) about 39 kb and 17.9 kb upstream of *ZmRap2.7* TSS, respectively. The *Vgt1*-IR construct was generated as follows: the regions of interest were amplified from B73 gDNA with primers containing attR sequences (primers in Supplementary Table S8) and cloned by a BP reaction into pDONR207 (Invitrogen). The cloned region was then transferred into the destination vector pBb7GW-I-WG-UBIL (https://vectorvault.vib.be/collection) (Karimi et al. 2007, 2013) by an LR reaction and the proper rearrangement of the construct and its sequence was verified by enzymatic restriction (*Bsr*GI) and by PCR followed by Sanger sequencing (primers in Supplementary Table S8). The generated expression clone was then transformed by heat shock into the hypervirulent *Agrobacterium* (*Agrobacterium tumefaciens*) strain EHA101 (Hood et al. 1986) and selected on YEB medium with kanamycin (50 mg/L) and spectinomycin (70 mg/mL).

Transgenic maize lines were generated in the B104 inbred line background by cocultivation of immature embryos with *Agrobacterium*, according to Coussens et al. (2012). Regenerated, rooted transgenic T0 shoots were selected from independently transformed immature embryos by a phosphinothricin acetyltransferase (PAT) enzyme assay (PAT is encoded by the bar selectable marker gene) and/or PCR genotyping, and crossed with B104 (T0 × B104 or B104 × T0) to generate heterozygous T1 seeds. Segregation ratios were determined by PAT assays on T1, T2, or T3 seedlings germinated in soil for about 1 wk. PAT proteins were detected with the TraitChek Crop and Grain Test Kit (Strategic Diagnostics), and PAT enzyme activity was analyzed with the ammonium multiwell assay (Rasco-Gaunt et al. 1999) with some modifications (Coussens et al. 2012). Alternatively, transgenic plants were distinguished from non-transgenic plants using PCR genotyping. DNA was prepared from a seedling leaf tip harvested approximately 1 wk after sowing. Samples were ground in 1.5 mL microcentrifuge tubes using micro pestles, 700 *μ*L of extraction buffer (100 mm Tris, pH 8, 50 mm EDTA, 500 mm NaCl) and 37 *μ*L of 20% SDS were added, followed by incubation for 20 min at 65 °C. Next, 187 *μ*L of 5 m potassium acetate was added and the samples mixed and incubated for 5 min on ice. The samples were spun for 5 min at max speed, and the supernatant precipitated by addition of 1 volume of isopropanol and 5 min centrifugation at max speed. Pellets were washed with 1 mL 70% EtOH. Ultimately, gDNA was resuspended in 50 *μ*L of 10 mm Tris, pH 7.5. To check for presence of the transgene, gDNA was PCR amplified with primers annealing to the BAR gene (primers in Supplementary Table S8) present in the T-DNA, followed by gel electrophoresis. For each line used in this study, segregation of the transgene in 50% of the T1 plants indicated the presence of one transgenic locus.

### Flowering time phenotyping

Flowering time was scored by measuring the number of DPS (first day of polled shed) and days to silking (first silks visible) and the total number of nodes (equal to the total number of leaves) in 3 independent experiments with 6 to 35 plants per line (Supplementary Fig. S2, A to C). Days were counted from the day of germination. Statistical analysis with ANOVA was used to identify significant differences from WT. The flowering time of plants used for the growth rate measurements was scored by counting days from stage V2 and using 10 to 24 plants per line. Raw data are provided in Supplementary Table S9. Apical meristems were carefully dissected under a binocular using forceps and then visualized using a Leica stereomicroscope with a microscope 1 mm calibration ruler.

### Growth rate phenotyping

Growth rate was measured for the non-transgenic lines N28, C22-4, and B104 and transgenic *Vgt1-*IR lines 327-38, 327-16, and 331-6 by determining the number of days to the various vegetative (V) growth stages for each plant (Fig. 2H; Supplementary Fig. S3A). The V stage was monitored daily and was defined by the number of visible leaf collars. For N28, C22-4, and B104, 15 seeds were sown, for IR lines 30 seeds (T2). All seeds were sown at the same time; of the *Vgt1*-IR lines, DNA was extracted from individual seedlings to test for transgene presence by PCR on the BAR gene. Non-transgenic seedlings were removed from the analysis. Each plant was assigned a t_0_ date corresponding to the date at which it reached stage V2 (leaf collar of leaf 2 visible). From this date, plants were scored daily and dates of reaching subsequent stages reported (V3 up to V21). To calculate the growth speed, the date at which each plant was reaching the next stage was subtracted from its t_0_ date and the results plotted (Supplementary Fig. S3B). Raw data are provided in Supplementary Table S10.

### Bisulfite sequencing

Tips of the second leaf of 3 to 4 plants per line were pooled (stage V2 for 327-38, stage V5 for all other lines) and ground in liquid nitrogen. Genomic DNA was extracted from the frozen powder using the DNeasy Plant Mini Kit (Qiagen, Cat. no. 69104). Bisulfite conversion was performed using the EpiTect Bisulfite Kit (Qiagen, Cat No. 59104). The conversion cycle program and the conversion efficiency check were performed as described in Foerster and Mittelsten Scheid (2010). Regions of interest were amplified using degenerated primers designed with the Kismeth webtool (Gruntman et al. 2008) (primers in Supplementary Table S8), cloned into the TOPO vector using the TA cloning system (Invitrogen). Plasmid inserts from at least 3 individual colonies were DNA sequenced using universal M13 reverse primers. Methylation analysis was performed using the webtool CyMATE (https://www.cymate.org/) (Hetzl et al. 2007).

### Reanalysis RNA-seq (Li et al. 2010)

We searched the AGPv4 gene names for *ZmRap2.7* (Zm00001d010987), *ZCN8* (Zm00001d010752), and *ZmMADS4* (Zm00001d034045) on the eFP browser (https://bar.utoronto.ca/transcriptomics/efp_maize/cgi-bin/efpWeb.cgi), data source “Maize leaf gradient” (Li et al. 2010), and mode “Absolute” to obtain a heat map of gene expression across 15 sections of the 3rd leaf of 9-d-old (V2 stage) B73 seedlings.

### RT-qPCR gene expression analysis

Total RNA was extracted from different plant tissues using TRizol. Then, 2 μg of RNA was treated with DNA-free DNase I (Roche, Cat. no. 04716728001), and cDNA was generated using the RevertAid First Strand cDNA Synthesis Kit and T18 oligonucleotide for priming (Thermo Scientific, Cat. no. K1622). Quantitative PCR was performed using 2 *μ*L of cDNA mixed with 2 *μ*L of each primer (10 *μ*M) and 4 *μ*L of the 5X FIREPol Evagreen qPCR Mix Plus (Solis Biodyne) in a total volume of 20 *μ*L and amplification on a real-time PCR cycler (Applied Biosystems QuantStudio3). Expression of *S-adenosyl methionine decarboxylase 2* (*SAM2*, Zm00001eb078440) and poly ubiquitin7 (*UBQ7*, GenBank: EU953010.1) was used to normalize *ZmRap2.7* (Zm00001eb355240), *ZCN8 (*Zm00001eb353250*)*, and *ZmMADS4* (Zm00001eb057540) expression levels using qBase (Hellemans et al. 2007). All primers are listed in Supplementary Table S8. Details of the analysis can be found in https://github.com/johanzi/scripts_zicola_vgt1. Raw data are provided in Supplementary Table S11.

### In situ hybridization

A detailed protocol for in situ hybridization is provided in the Supplementary methods. The protocol is mainly based on a protocol developed for petunia floral buds by Marco Busscher and Gerco Angenent (CPRO-DLO, Wageningen, the Netherlands; added to GitHub [https://github.com/KoesGroup/Protocols/blob/master/In_Situ_Hybridisation.md] by Ronald Koes group [University of Amsterdam, the Netherlands]). Parts of the protocol were based on a published protocol (Javelle and Timmermans 2012). To generate a *ZmRap2.7* probe, a nested PCR was performed on cDNA generated as described above (qPCR gene expression analysis) using RNA from the above ground tissue of stage V3 B104 seedling, with primers M3197 (GAAGTAGACACCACCAGTTCGC) and M3212 (CAGGCCTCCTGTGGAGATCT), followed by M3247 (GGTAGTTAGCAGCCCAGTTGC) and M1465 (TGCACTCATCTCATGGTTTGT). Both reactions were 30 cycles, using DreamTaq (Fisher Scientific EP0702). One nanogram of the first PCR was used for the second reaction. The 1240 bp PCR fragment was cleaned using the GENEJET Plasmid Minirep kit (Thermo Fisher K0503) and ligated into pGEM-T Easy (Promega) according to manufacturer's instructions.

### RNA-seq expression analysis

Total RNA was isolated using TRIzol (Invitrogen) from parts B and D of leaf 4 at stage 3 (V3L4) from 4 individual plants for B104 WT and 2 *Vgt1-*IR lines. 3′-Digital gene expression (3′-DEG) libraries were prepared with the BrAD-seq protocol (Townsley et al. 2015). The 24 barcoded libraries were pooled and sequenced by BGI on the MGI2000 sequencer in mode PE50 + 5 + 10. About 6.7 to 20.4 million reads were obtained per library. Untrimmed reads were mapped on the B73 NAM5 reference genome (release 51) with HISAT2 (2.1.0) (Kim et al. 2019). Read count for each transcript was calculated using HTseq (0.6.1) (Anders et al. 2015) using the B73 NAM5 transcript annotation (release 51) from the Ensembl server (http://ftp.ensemblgenomes.org/pub/plants/release-51/gtf/zea_mays/Zea_mays.Zm-B73-REFERENCE-NAM-5.0.51.gtf.gz  ftp://ftp.ensemblgenomes.org/pub/plants/release-51/gtf/zea_mays/Zea_mays.Zm-B73-REFERENCE-NAM-5.0.51.gtf.gz). Raw read counts for each sample were merged into one matrix using a home-made bash script. The raw read count matrix was imported in R (4.0.3) and processed using DESeq2 (1.28.1) for PCA and gene expression analysis (Love et al. 2014). For the GO term enrichment analysis, we used a published annotation for B73 NAM5 generated using the GOMAP pipeline (Wimalanathan and Lawrence-Dill 2021; Fattel et al. 2024 ) and perform the GO enrichment analysis using the clusterProfiler R package (Wu et al. 2021). Details on the GOMAP pipeline can be found on https://github.com/johanzi/GOMAP_maize_B73_NAM5. Details of the RNA-seq analysis can be found in https://github.com/johanzi/scripts_zicola_vgt1.

### ChIP-seq for ZmRap2.7

The coding sequence of *ZmRap2.7* (Zm00001eb355240_T002, https://www.maizegdb.org/gene_center/gene/Zm00001eb355240), encoding a 475 amino acids protein, was cloned into the p35S-GW-3Avi vector (Addgene #127918) using the Ready-to-Use Seamless Cloning Kit (Sangon) (primers listed in Supplementary Table S8). Protoplast transformation and library preparation were performed exactly as described in Tu et al. (2020). Briefly, we transformed the 35S::Rap2.7-3×Avi construct alongside the pBirA-P19 plasmid (Addgene #127919) into protoplasts from 9-d-old B73 seedling leaves using polyethylene glycol. Subsequently, ChIPmentation libraries were prepared and sequenced on the Illumina HiSeq X platform in paired-end mode (150 + 150). The raw sequencing data of ZmRap2.7 libraries and the input control published previously (Tu et al. 2020) were used to determine the binding site motifs (Supplementary Table S12). Raw reads were processed using fastp software (v0.23.2) with default parameters to trim adapter sequences and eliminate low-quality reads. Trimmed reads were aligned to the 10 chromosomes of the B73 NAM5 reference genome using Bowtie2 (v2.5.0) (Langmead and Salzberg 2012) with default parameters. Unmapped and unpaired reads were removed with samtools (version 1.15.1) (samtools view, parameters -F 4 -f 3), and PCR duplicates were removed using the rmdup function. Peak calling was performed with GEM (v3.4) (Guo et al. 2012) (parameters –k_min 4 –k_max 12 –q 3 –fold 3 –nrf –outNP). NarrowPeak files generated from the 2 biological replicates were analyzed using the IDR (Irreproducible Discovery Rate) package (version 2.0.4.2) (Li et al. 2011) with a significance threshold of 0.01 (–idr-threshold 0.01). For binding motif analysis, the replicable peaks from each biological replicates were retrieved with samtools (bedtools intersect -a peak_rep[1|2].bed -b idr_peak.txt -wa -u > rep[1|2]_passed_IDR.bed). Only the overlapping peaks from the first replicate were then selected (bedtools intersect -a rep1_passed_IDR.bed -b rep2_passed_IDR.bed -wa -u > peak_final.bed). The peaks overlapping the regions of the TF analyzed were removed (likely to be residual plasmid sequenced) (bedtools intersect -v -a peak_final.bed -b TF_gene_coordinates.bed > peak_final.cleaned.bed). The fasta sequence for each peak was retrieved using samtools (fastaFromBed -fi Zm_NAM5.fa -bed peak_final.cleaned.bed -fo peak_final.cleaned.fa). The top 1000 peaks were analyzed with MEME-CHIP (v5.5.5) (Machanick and Bailey 2011) using a motif window of 8-nt maximum and the database of Arabidopsis motifs previously published (Franco-Zorrilla et al. 2014) (meme-chip -dna -db motif_databases/ARABD/ArabidopsisPBM_20140210.meme -maxw 8 -o output_name -meme-mod zoops -spamo-skip -fimo-skip rep1_passed_IDR.fa). Visualization of ZmRap2.7 and other EREB TF binding motifs was performed with the motifStack R package (version 1.32.1) (Ou et al. 2018) using as input the significant motifs from the MEME-CHIP output file “combined.meme”. Details of the analysis can be found in https://github.com/johanzi/scripts_zicola_vgt1.

### 4C-seq

Three biological replicates were prepared per tissue type: inner stem tissue (IST) of V2 seedling (V2-IST) and husk from B73 inbred line plants. First 3C samples were generated as described (Louwers et al. 2009; Weber et al. 2018). For each biological replicate, IST from 5 V2 seedlings or the soft (non-lignified) leaves from one husk were cross-linked for 1 h with 2% formaldehyde in phosphate sodium buffer and used to isolate nuclei. Chromatin was then extracted and digested using 400 units of *Bgl*II (A/GATCT) restriction enzyme per sample. Fixed and digested chromatin was subsequently submitted to an intra-molecular ligation step using 100 units of T4 DNA ligase, and ligated fragments were recovered after de-crosslinking and precipitation. The quality of chromatin prior to digestion, after digestion, and after ligation was evaluated for each sample on an 0.8% Agarose 0.5× TAE gel. To generate 4C-seq libraries, the 3C samples were submitted to a second round of digestion and ligation basically as described (Splinter et al. 2012) with minor modifications. The entire 150 *μ*L of each 3C sample was digested overnight at 37 °C using 50 units of *Csp*6I (G/TAC; ER0211-ThermoScientific), 1× Blue Digestion Buffer in a final volume of 500 *μ*L. Digestion efficiency was evaluated on a 0.6% Agarose 0.5× TAE gel by comparing 5 *μ*L aliquots to unfixed gDNA digested with *Csp*6I. If properly digested, *Csp*6I was heat inactivated (20 min at 65 °C). Digested DNA was then ligated overnight at 16 °C in a total volume of 1.4 mL using 100 units of T4 DNA ligase. Next, DNA was precipitated using 1/10 volume of 2 m NaOAc pH 5.6, 1/1,000 volume of glycogen, 2 volumes of 96% ethanol, and an incubation time of 2 h at −80 °C. DNA was recovered upon centrifugation (1 h, 4500 rpm, 4 °C). The resulting pellets were washed with 15 mL of 70%, spun again (30 min, 4500 rpm, 4 °C), and DNA was eluted for 1 h at 37 °C in 150 *μ*L of 10 mm Tris pH 7.5 to ensure complete resuspension of the DNA. Finally, samples were column purified (QIAquick PCR purification kit, Qiagen) using 3 columns per sample. For each column, elution was achieved with 2 successive centrifugations (first 35 *μ*L and then 20 *μ*L Elution Buffer) and eluates from columns belonging to the same sample were pooled together. To check the quality of the obtained 4C samples, 5 *μ*L of each sample was loaded on a 0.6% agarose TAE gel and compared to unfixed gDNA digested with *Csp*6I. Sample concentrations were measured by Qubit (Thermo Fisher); 4C-seq primers were designed for the ZmRap2.7 TSS region (chr8:135,653,433-135,654,281) as viewpoint, as close as possible to the primary (RE1: *Bgl*II) and secondary (RE2: *Csp*6I) restriction sites on either side of the viewpoint. The primers were blasted against the *Z. mays* B73 AGPv4 reference genome and only primers with one perfect match were kept. Finally, the specificity of each primer was evaluated through PCR amplification. For this, PCRs were prepared as described in the above section, using both primers (RE1 + RE2), one primer (RE1 or RE2), or no primer, and 2 different types of template: 30 ng of 4C sample or B73 gDNA. Only primers generating no amplification product when used alone and giving a specific amplification pattern when used as a pair on 4C template were selected to prepare viewpoint-specific 4C samples through PCR amplification (Supplementary Table S8). For each 4C sample, 16 PCRs were prepared (for 1 reaction, 30 ng of 4C DNA, 1 *μ*L 50 mm MgCl2, 1 *μ*L 10 *μ*M Fwd primer, 1 *μ*L 10 *μ*M Rev primer, 10 *μ*L KAPA HIFI 2 × readymix, miliQ up to 20 *μ*L) and amplified as follow: 2′, 98 °C; 30 × (15″, 98 °C; 15″, 60 °C; 3′, 72 °C); 5′, 72 °C; 12 °C. PCRs were then column purified using 1 column for 8 pooled PCRs and 25 *μ*L of elution buffer (QIAquick PCR purification kit, Qiagen). The 2 eluates per 4C sample were pooled (final volume = 50 *μ*L). Finally, viewpoint-specific 4C samples were converted into 4C-seq samples using Illumina Truseq sequencing adapters (with index), following the instructions from the KAPA Hyper prep kit for Illumina sequencing. For sequencing, all 4C-seq libraries were pooled together in equimolar condition (1 mm each) in a final volume of 10 *μ*L and sequenced on a Hiseq Illumina sequencer in 125 bp single-end mode. To increase the diversity of nucleotides in the beginning of each read, 20% PhiX genomic DNA was spiked in. Two V2-IST biological replicates yielded low amount of reads and were resequenced on a Hiseq Illumina sequencer in 150 bp paired-end mode.

Raw fastq files were processed with the pipe4C pipeline (Krijger et al. 2020). First, a Biostring object for the 10 chromosomes of the maize genome B73 NAM5 was generated with the R BSgenome package (v1.72.0). Then, all 6 4C-seq libraries were processed in pipe4C in “cis” mode (Rscript pipe4C.R –vpFile=VPinfo.txt –fqFolder=/path/to/fastq –outFolder=/path/to/output/ –wig). For each library above 90% of reads mapped within 1 Mb of the view point (see column cov1Mb in Supplementary Table S13). Significant peaks for each tissue were called using the peakC function from the pipe4C pipeline and the genomic coordinates were included in Supplementary Table S6 in Browser Extensible Data format. Details of the analysis can be found in https://github.com/johanzi/scripts_zicola_vgt1.

### Accession numbers

Sequence data from this article can be found in the GenBank/EMBL data libraries under accession numbers PRJNA1110215 (RNA-seq) and PRJNA1110214 (4C-seq). The MaizeGDB Gene IDs of the main genes in this paper are listed between brackets: *ZmRap2.7* (Zm00001eb355240), *ZCN8* (Zm00001eb353250), *ZmMADS4* (Zm00001eb057540).

## Supplementary Material

kiaf468_Supplementary_Data

## Data Availability

Raw sequencing data are available in NCBI for RNA-seq (PRJNA1110215, https://dataview.ncbi.nlm.nih.gov/object/PRJNA1110215?reviewer=s7k2g2jl2o8qa8pvkftlsj5kpb), ZmRap2.7 ChIP-seq (PRJNA1110977, https://dataview.ncbi.nlm.nih.gov/object/PRJNA1110977?reviewer=mmk4go2ku58ffdatdq7b5jib9n), and 4C-seq data (PRJNA1110214, https://dataview.ncbi.nlm.nih.gov/object/PRJNA1110214?reviewer=s3qmviph27vq6bfllph5p59epm). Scripts of all analyses are available on the GitHub repository https://github.com/johanzi/scripts_zicola_vgt1.

## Supplementary data

The following materials are available in the online version of this article.

**Supplementary Figure S1.** Representation of the *Vgt1* locus and details of the *Vgt1*-IR construct.

**Supplementary Figure S2.** Flowering time measurements of *Vgt1*-IR transgenic lines across 3 independent experiments.

**Supplementary Figure S3.** Growth speed and flowering time analysis of *Vgt1*-IR lines compared to various non-transgenic maize accessions.

**Supplementary Figure S4.** Maize meristems during the reproductive transition and *ZmRap2.7* expression.

**Supplementary Figure S5.** Expression analysis of marker genes across developmental stages by RT-qPCR.

**Supplementary Figure S6.** Expression of *ZCN8* in part B and D of leaves in WT and 2 *Vgt1*-IR transgenic lines.

**Supplementary Figure S7.** Identification of TF binding sites in ZmRap2.7 ChIP-seq data.

**Supplementary Figure S8.** GO term enrichment for ZmRap2.7-bound genes and detailed binding profile at *ZCN8.*

**Supplementary Figure S9.** Chromosomal interactions at the*Vgt1*-*ZmRap2.7* locus using 4C-seq.

**Supplementary Figure S10.** H3K27me3 levels at *Vgt1* and DMR in leaf tissue and ear primordia.

**Supplementary Table S1.** DEGs between *Vgt1*-IR and B104 WT—leaf part B and D.

**Supplementary Table S2.** GO term enrichment analysis B104 WT vs *Vgt1*-IR DEGs.

**Supplementary Table S3.** ZmRap2.7 ChIP-seq peaks.

**Supplementary Table S4.** Differentially expressed ZmRap2.7 target genes.

**Supplementary Table S5.** GO term enrichment analysis of ZmRap2.7 target genes.

**Supplementary Table S6.** 4C-seq peaks for V2-IST and husk.

**Supplementary Table S7.** Genomic coordinates *Vgt1* and CNSs.

**Supplementary Table S8.** List of oligonucleotides.

**Supplementary Table S9.** Flowering time data.

**Supplementary Table S10.** Growth rate data.

**Supplementary Table S11.** RT-qPCR data for *ZmRap2.7*, *ZCN8*, and *ZmMADS4.*

**Supplementary Table S12.** Details of ChIP-seq data.

**Supplementary Table S13.** 4C-seq statistics report.

**Supplementary methods.** RNA in situ hybridization using digoxigenin labeled RNA probes.
