# Supplementary material for: Vegetative to generative1 (Vgt1) is an enhancer affecting flowering time and jasmonate signaling in maize by promoting the expression of Zea mays Related to APETALA 2.7
Source: Plant Physiol. 2025 Oct 3;199(3):kiaf468. doi: 10.1093/plphys/kiaf468 (PMC12610936; doi:10.1093/plphys/kiaf468)
Supplement: kiaf468_Supplementary_Data [file kiaf468_supplementary_data.zip › all_supp_figures_merged.pdf]

A

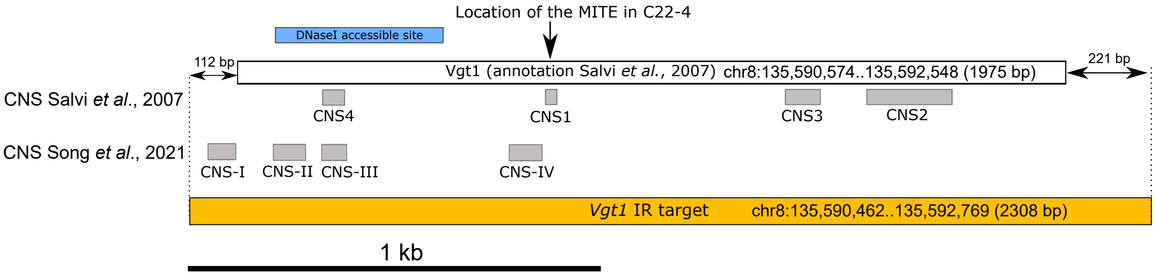

B

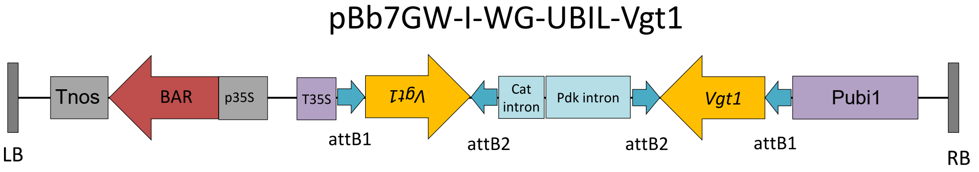

Supplementary Figure 1: (**A**) Annotation of *Vgt1* as defined in Salvi *et al.,* 2007, with the conserved non-coding sequences (CNS) shared with rice and sorghum (Salvi et al. 2007) and the CNS shared with Sorghum (Song et al. 2021) indicated by grey boxes, the location of the MITE insertion in C22-4, and the region targeted by the IR, encompassing *Vgt1*. The blue square indicates a DNaseI accessible site found in V2 seedling inner stem tissue (Oka et al 2017). Genome coordinates given for Zm-B73-REFERENCE-NAM-5.0 genome assembly (see BED coordinates in **Supplementary Table 13**). (**B**) Detailed representation (not on scale) of *Vgt1*-IR construct used for Agrobacterium-mediated transformation of the B104 maize line. The *Pubi1:Vgt1-1tgV* IR construct was generated in a pDONR vector and subsequently transferred to the Bb7GW-I-WG destination vector using a Gateway LR reaction. Pubi1 refers to the maize *ubi1* gene promoter (GRMZM2G409726). P35S (1028 bp) and T35S refer to the Cauliflower Mosaic Virus 35S RNA gene promoter and terminator, respectively. Blue arrows indicate Gateway cloning sites. Tnos refers to the terminator sequence of the *nopaline synthase* (*nos*) gene from *Agrobacterium tumefaciens*. The cat intron and Pdk intron refer to the introns of the *chloramphenicol acetyltransferase* and *pyruvate dehydrogenase kinase* genes, respectively. LB and RB indicate the left and right T-DNA border, respectively.

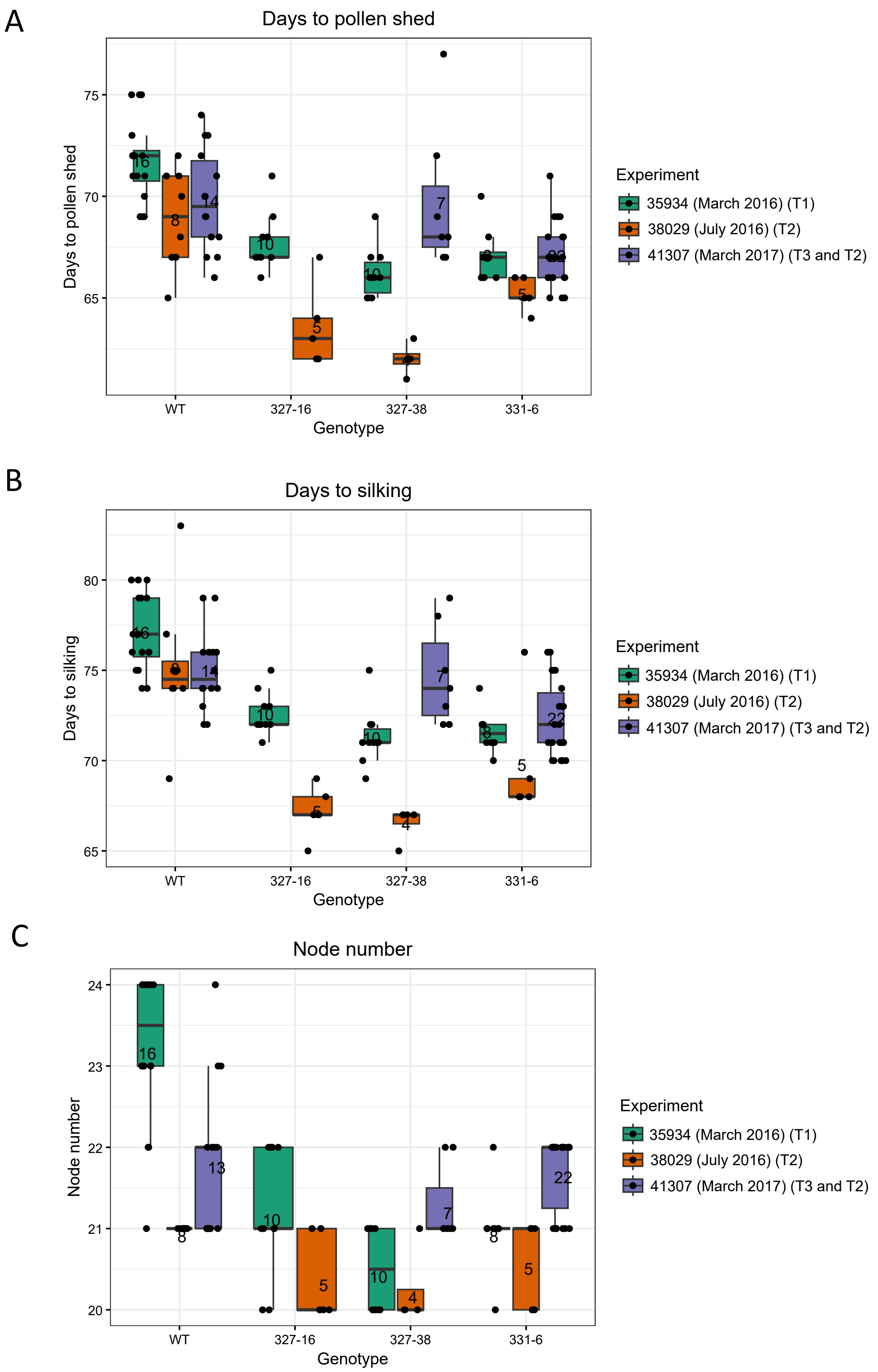

Supplementary Figure 2: Flowering time measurements of *Vgt1*-IR transgenic lines across three independent experiments. Days to pollen shed (**A**), days to silking (**B**) and node number (**C**). Center lines of box plots show the medians, box limits the 25th and 75th percentiles, whiskers extend 1.5 times the interquartile range from the 25th and 75th percentiles, dots represent individual plants. Culture identifiers (5-digit number), month of sowing, and generation of transgenic plants used are specified in the plot legend. 4 to 22 replicates were used by experiment and genotype.

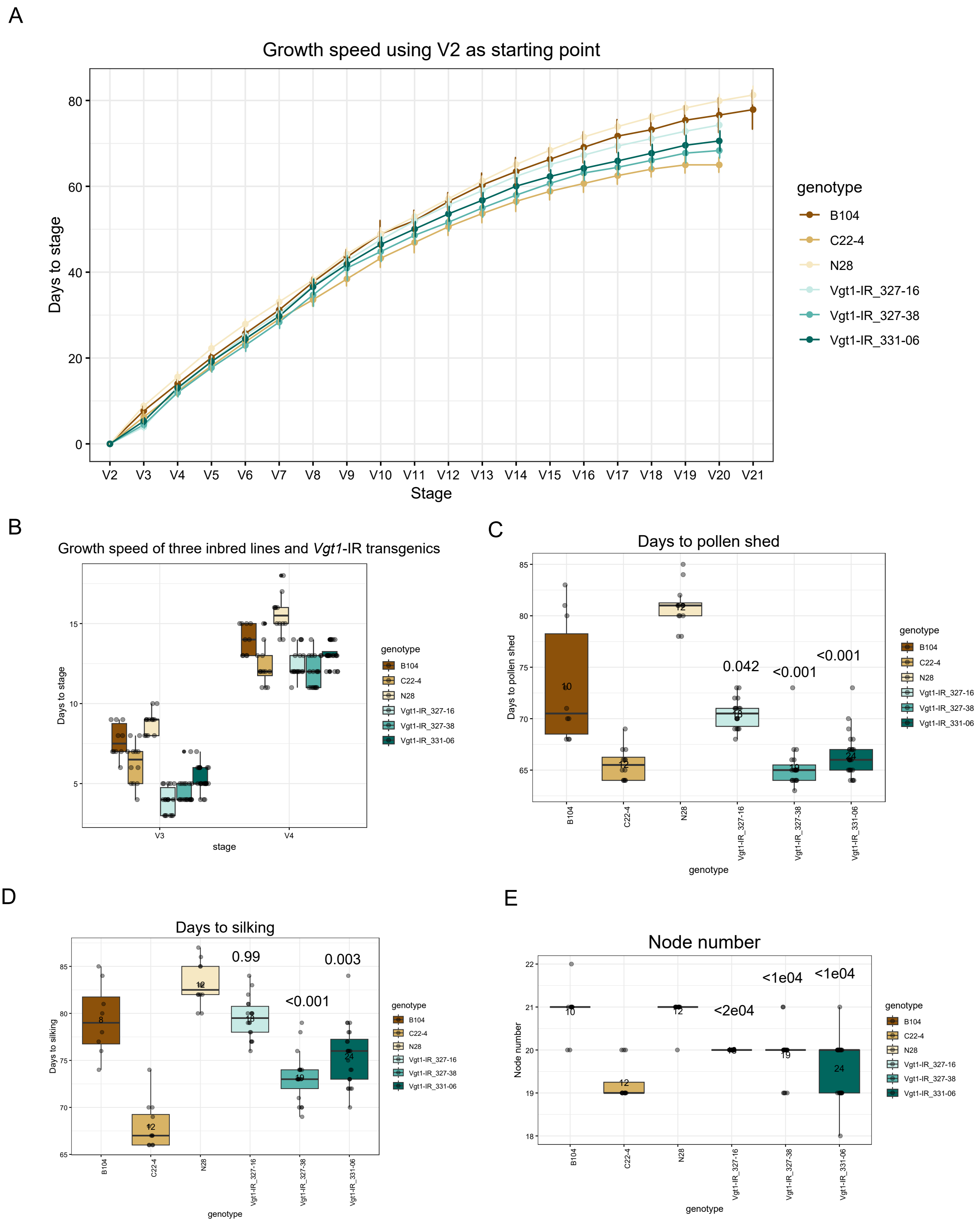

Supplementary Figure 3: Growth speed analysis and flowering time of *Vgt1*-IR lines compared to N28, C22-4 and B104 lines. (**A**) Growth speed analysis of B104 and transgenic *Vgt1*-IR lines (327-16, 327-38 and 331-06) in comparison with late and early flowering lines N28 and C22-4, respectively. The plot shows the number of days each line on average took to reach the developmental stages indicated (V3 to V21), starting from the V2 stage. The steeper the line between two stages, the slower the growth rate. Error bars represent the standard error of the mean for 10 to 24 biological replicates (Number of plants per line: N28 = 12; C22-4 = 12; B104 = 10; Vgt1-IR_327-16 = 18; Vgt1-IR_327-38 = 19; Vgt1-IR_331-06 = 24). (**B**) Number of days to stage V3 and V4 for all genotypes. (**C**-**E**) Flowering time for plants analyzed in (**A**) and (**B**) expressed in days to pollen shed (DPS) (**C**), days to silking (**D**), and node number (**E**). ANOVA followed by a Dunnett's test (two-sided) was performed to compare each *Vgt1*-IR transgenic line to B104. P-values are indicated on the plots.

A

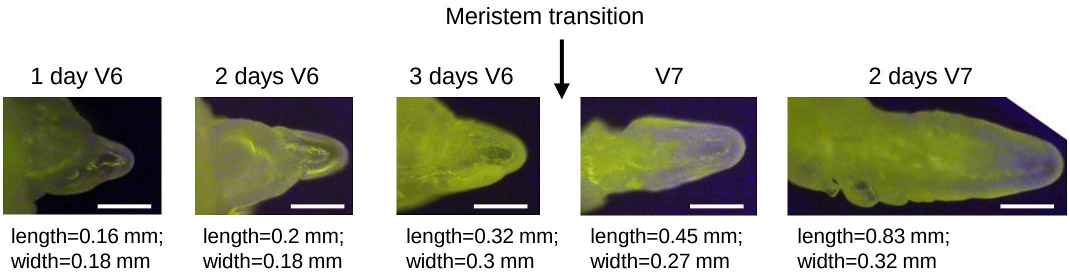

B

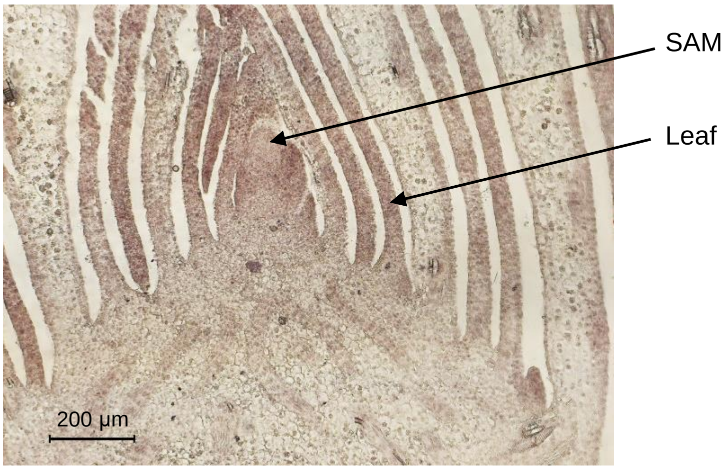

Supplementary figure 4. Meristems during floral transition and *ZmRap2.7* expression. (**A**) pictures of B104 shoot apical meristems (SAMs) at the developmental stages indicated. During the floral transition, the meristem starts elongating, which can be observed at the V6 stage (Irish and Nelson et al, 1991). In the V7 stage branches started appearing on the flanks of the SAM. (**B**) *In situ* hybridization of the shoot apical meristem of B104 V3 plants (11 days staining, 10x magnification) reveals *ZmRap2.7* transcripts in leaf primordia, leaf tissue and vasculature, but not the SAM.

Reference:

Irish EE, Nelson TM. **1991**. Identification of multiple stages in the conversion of maize meristems from vegetative to floral development. *Development* **112**: 891–898.

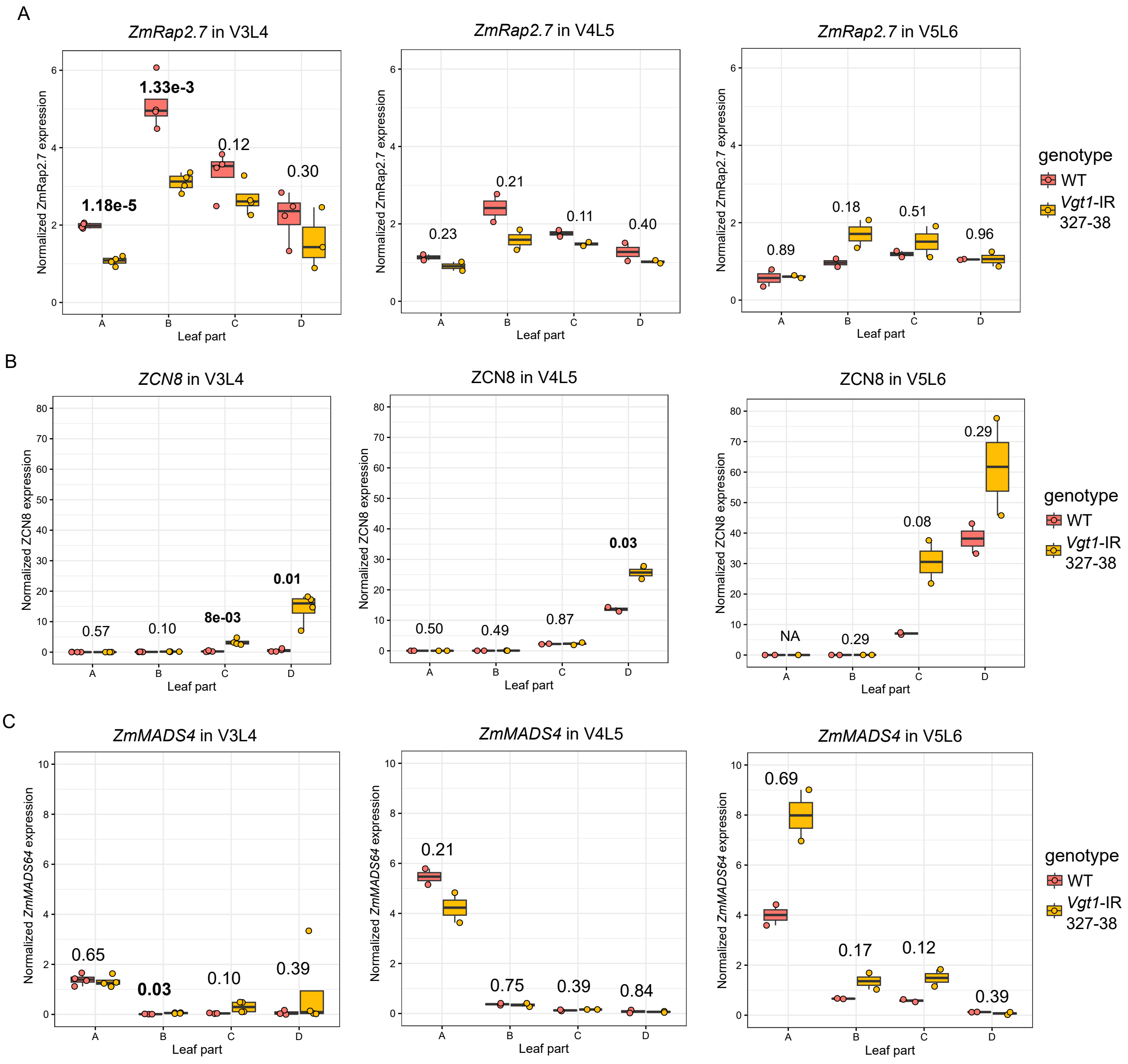

Supplementary Figure 5: qPCR expression for *ZmRap2.7* (**A**), *ZCN8* (**B**), and *ZmMADS4* (**C**) in leaf parts of three maize developmental stages for WT and the *Vgt1*-IR line 327-38. Four biological replicates were used for stage V3L4 tissue and two biological replicates were use for stage V4L5 and V5L6. T-tests or Welch's tests were performed based on variance equality between the genotypes (two -tailed test). P-values are indicated on the plots and put in bold when significant (below 5%). No p-value could be calculated for *ZCN8* expression in V5L6 leaf part A because one group contains only one replicate (NA on the plot). Center lines of box plots show the media ns, the box limits the 25th and 75th percentiles, whiskers extend 1.5 times the interquartile range from the 25th and 75th percentiles, dots represent individual data points.

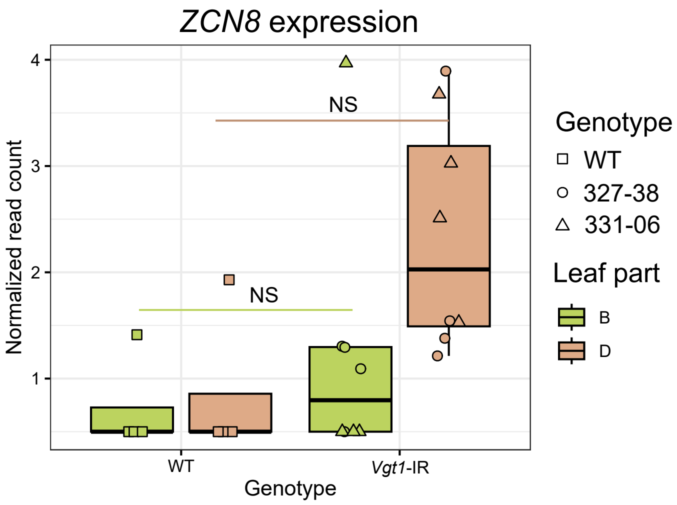

Supplementary Figure 6: Expression of *ZCN8* in part B and D from WT and two *Vgt1*-IR transgenic lines. Expression of *Vgt1*-IR lines and WT were compared for each leaf part and did yield not significant differences (NS) using the Wald test from the DESeq2 R package. Center lines of box plots show the medians, box limits the 25th and 75th percentiles, whiskers extend 1.5 times the interquartile range from the 25th and 75th percentiles, squares, dots, and triangles represent individual data points.

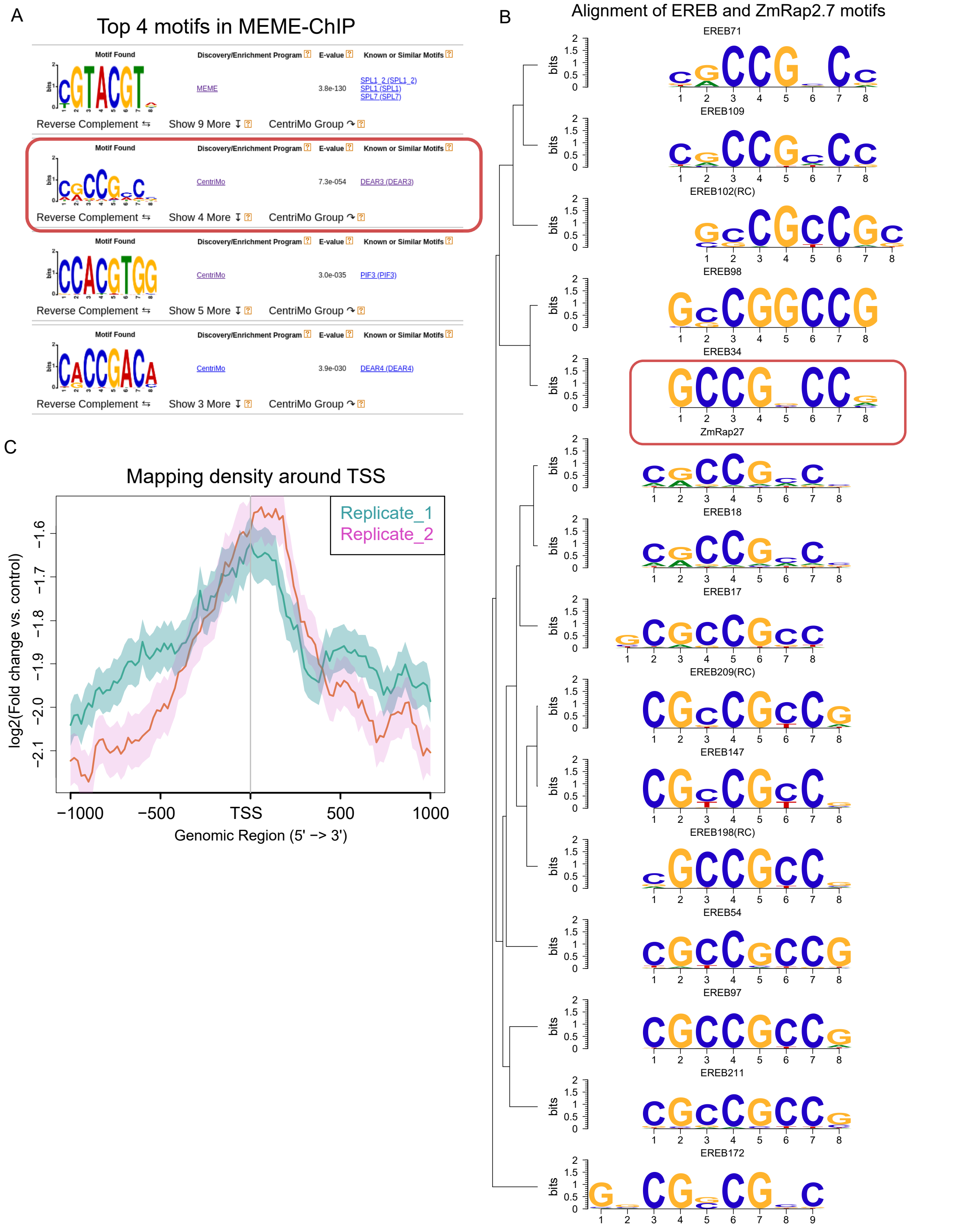

Supplementary Figure 7: (**A**) Snapshot of the MEME-ChIP report for the top 5 motifs. The CGCCGCCG motif highlighted in red was then used for comparison with the top motifs of EREB TFs in (**B**). (**B**) Hierarchical clustering tree of the top motif of 14 different EREB TFs and the second motif of ZmRap2.7 (boxed in red). y-axis represents information content in bits. (**C**) Mapping density normalized by input control for the two biological replicates at transcription start sites (TSS).

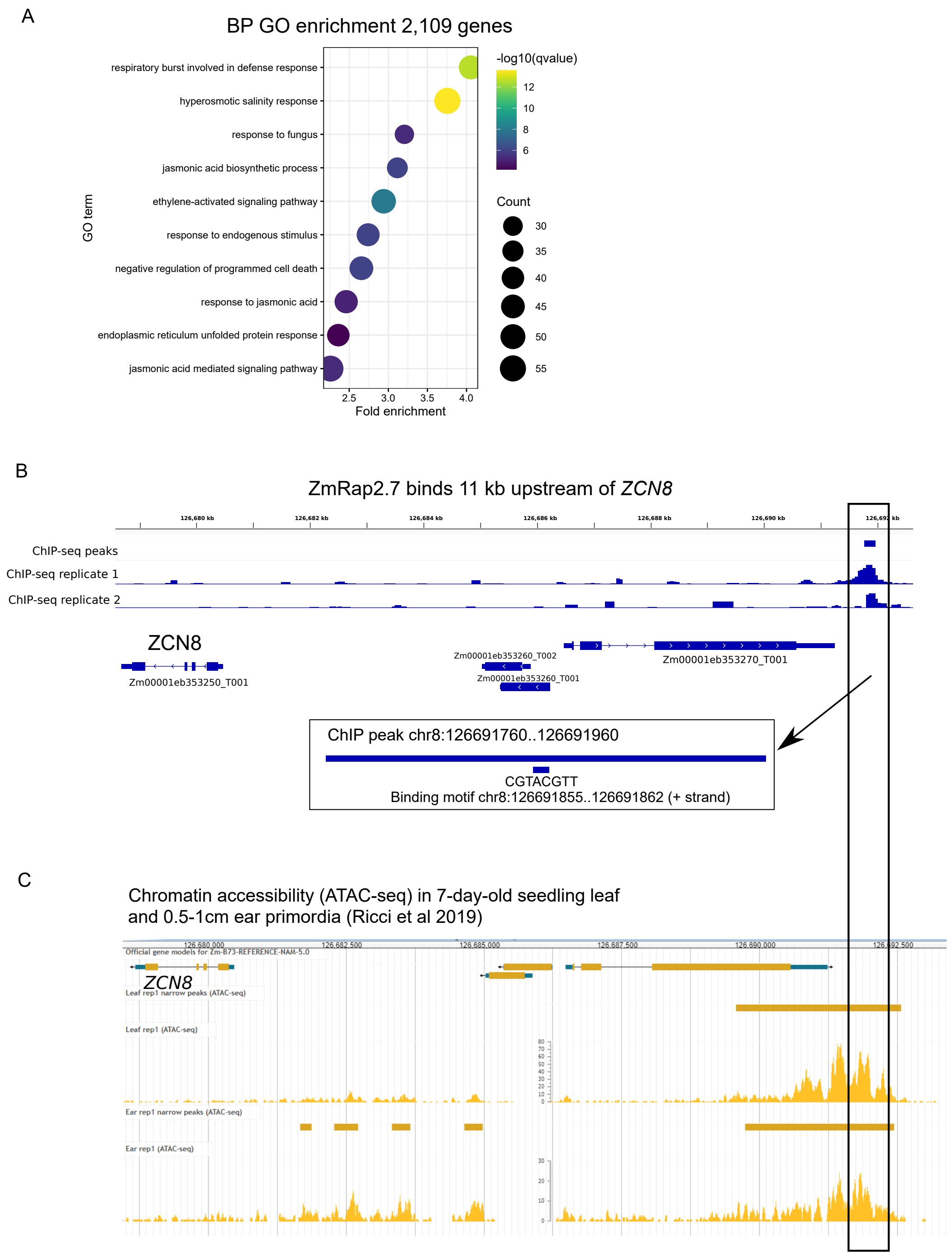

Supplementary Figure 8: (**A**) GO enrichment analysis for biological processes performed for the 2,109 genes containing ChIP-seq peaks from 10 kb upstream of the TSS to 5 kb downstream of the TTS. Only the top ten GO terms with the lowest q-values are displayed, full GO results are in **Supplementary Table 5**. (**B**) Location of the ChIP-seq peak 11 kb upstream of *ZCN8* (Zm00001eb353250). The peak is indicated by a rectangle, while the two tracks beneath represent the normalized read counts for each replicate of the ZmRap2.7 ChIP-seq. Genes are indicated on the bottom track. Inset defines the coordinates of the ChIP-seq peak and the CGTACGTT binding motif identified as top motif in the MEME analysis. **(C)** Chromatin accessibility data (ATAC-seq) from for 7-day-old seedling leaf and 0.5-1 cm ear primordia (Ricci et al. 2019). The Black square indicates the region bound by ZmRap2.7.

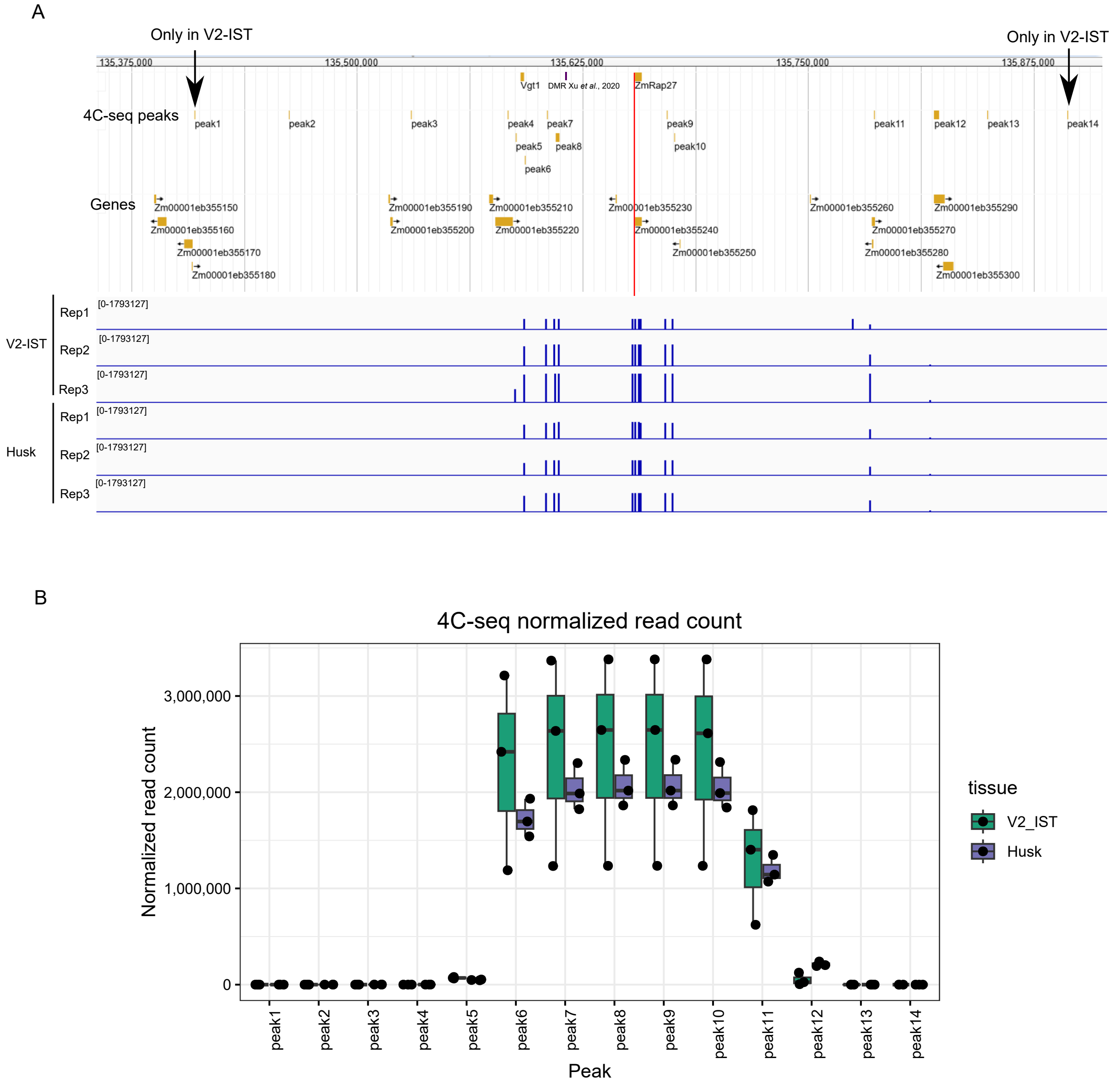

Supplementary Figure 9: (**A**) Chromosomal interactions in a 500 kb region encompassing *ZmRap2.7* in V2-IST and husk tissue using the TSS region of *ZmRap2.7* as viewpoint (chr8:135,653,433-135,654,281, red line). *Bgl*II restriction sites with significant levels of interaction (peaks) are numbered from 1 to 14, peak 1 and peak 4 being significant only in V2 -IST (indicated by black arrows). Blue vertical bars indicate the normalized read count at *Bgl*II restriction sites for each replicate and tissue type (range of values is given in square brackets). (**B**) Normalized read count for each peak in both tissues. Center lines of box plots show the medians, box limits the 25th and 75th percentiles, whiskers extend 1.5 times the interquartile range from the 25th and 75th percentiles, dots represent individual biological replicates.

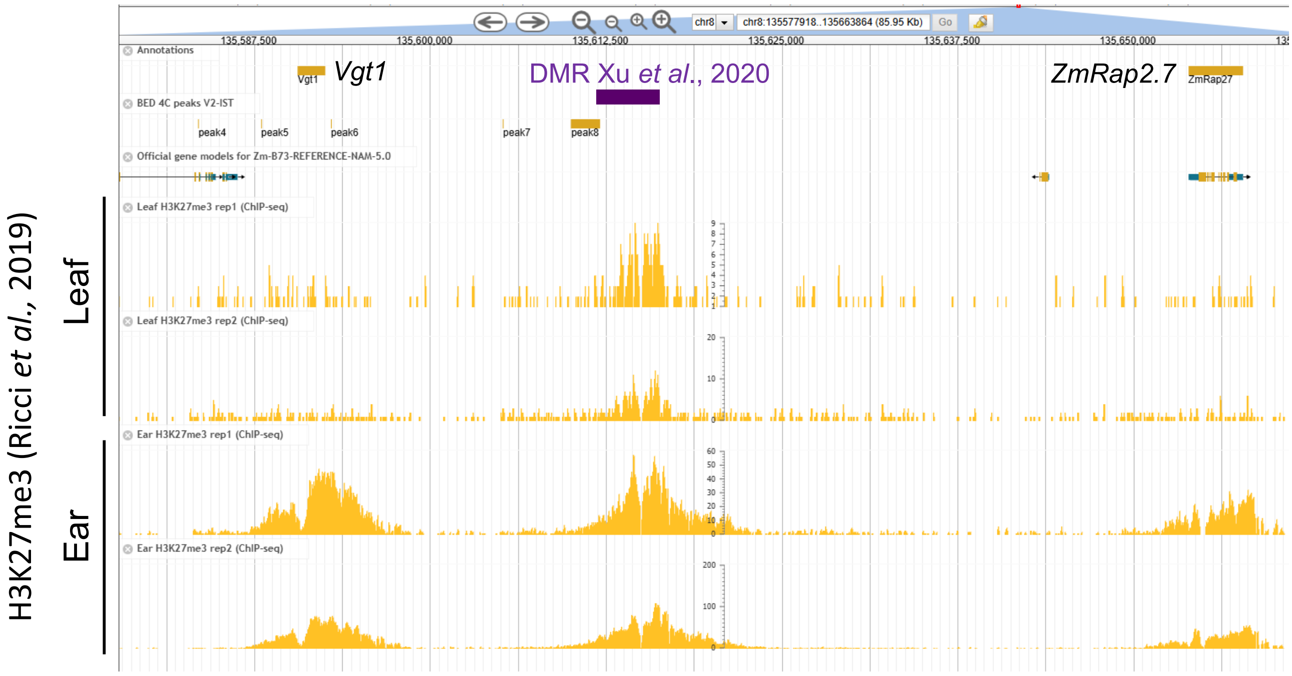

Supplementary Figure 10: H3K27me3 levels at Vgt1 in leaf (6-day-old seedlings) and ear primordia for two replicates (Ricci et al. 2019), and DMR (Xu et al. 2020). Snapshot from https://jbrowse.maizegdb.org/. Y-axis values for H3K27me3 vary across tracks. 4C-seq peaks for IST-V2 are indicated.
