# Supplementary material for: Vegetative to generative1 (Vgt1) is an enhancer affecting flowering time and jasmonate signaling in maize by promoting the expression of Zea mays Related to APETALA 2.7
Source: Plant Physiol. 2025 Oct 3;199(3):kiaf468. doi: 10.1093/plphys/kiaf468 (PMC12610936; doi:10.1093/plphys/kiaf468)
Supplement: kiaf468_Supplementary_Data [file kiaf468_supplementary_data.zip › supp_Figure_4_in situ_meristems[76].pdf]

A

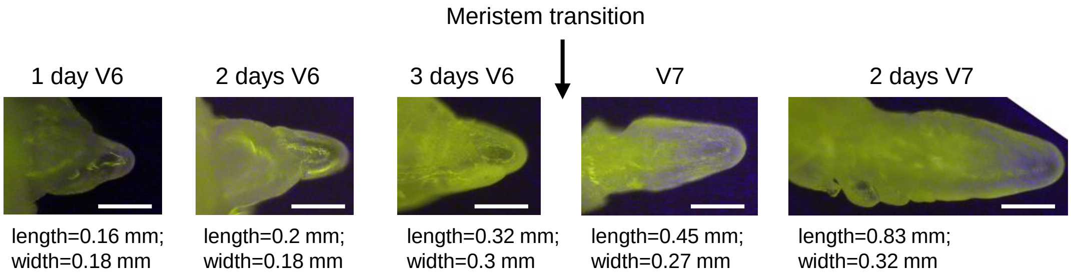

B

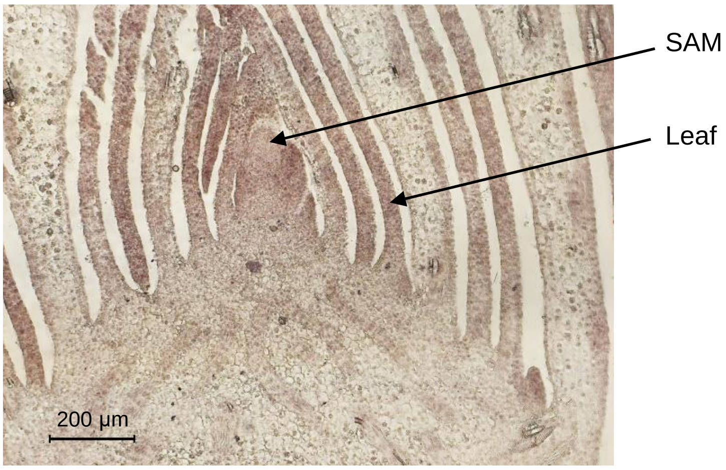

Supplementary figure 4. Meristems during floral transition and *ZmRap2.7* expression. (**A**) pictures of B104 shoot apical meristems (SAMs) at the developmental stages indicated. During the floral transition, the meristem starts elongating, which can be observed at the V6 stage (Irish and Nelson et al, 1991). In the V7 stage branches started appearing on the flanks of the SAM. (**B**) *In situ* hybridization of the shoot apical meristem of B104 V3 plants (11 days staining, 10x magnification) reveals *ZmRap2.7* transcripts in leaf primordia, leaf tissue and vasculature, but not the SAM.

Reference:

Irish EE, Nelson TM. **1991**. Identification of multiple stages in the conversion of maize meristems from vegetative to floral development. *Development* **112**: 891–898.
