# Supplementary material for: Vegetative to generative1 (Vgt1) is an enhancer affecting flowering time and jasmonate signaling in maize by promoting the expression of Zea mays Related to APETALA 2.7
Source: Plant Physiol. 2025 Oct 3;199(3):kiaf468. doi: 10.1093/plphys/kiaf468 (PMC12610936; doi:10.1093/plphys/kiaf468)
Supplement: kiaf468_Supplementary_Data [file kiaf468_supplementary_data.zip › Zicola-supplementary_methods.pdf]

Supplementary methods: RNA in situ hybridization using digoxigenin labeled RNA probes

Contents

A) Fixation of the material: 1
B) Embedding in Paraplast (paraffine chips): 1
C) Sectioning and treatments of the tissue 2
D) Synthesis of digoxigenin labeled RNA 3
E) Hybridization of the slides 3
F) Washing 4
G) Immunological detection: 4

This protocol is a compilation of protocols from Marco Busscher and Gerco Angenent (WUR, Wageningen) and Javelle and Timmermans (2012). Modified from https://github.com/KoesGroup/Protocols/blob/master/In_Situ_Hybridisation.md

Use DEPC-treated Milli-Q for all solutions, except Tris. Tris cannot be autoclaved in DEPC-treated water. Mix autoclaved Tris with DEPC Milli-Q.

A) Fixation of the material:

The fixation is done at 4°C during 2 days. Fixative is not very stable for a long time so make fresh!

Fixative Mix (EAF) per 100 ml: 50 ml Ethanol absolute.

- 5 ml Acetic Acid
- 10 ml Formaldehyde 37%
- 35 ml Milli-Q Water
- Place plant material (meristems) in a 50 ml Greiner tube filled with 20 ml EAF.
- Pull vacuum for 15 minutes, start very slowly. Release the vacuum also very slowly.
- Incubate 1 hour at 4°C in EAF.
- Repeat vacuum step and incubation step 3 (three) times and leave for 2 days at 4˚C, with the caps on the tubes.

B) Embedding in Paraplast (paraffine chips):

- Remove EAF and rinse with DEPC-water (0.1% DEPC; 2 hrs 37˚C; autoclave at 120˚C)
- Dehydrate 30 minutes 70% Ethanol, Room Temperature (RT)
- Dehydrate 30 minutes 96% Ethanol, RT.

- Dehydrate 30 minutes 100% Ethanol, RT.
- Incubate 30 minutes in 75% Ethanol, 25% Xylene, RT.
- Incubate 30 minutes in 50% Ethanol, 50% Xylene, RT.
- Incubate 30 minutes in 25% Ethanol, 75% Xylene, RT.
- Incubate 3 times 15 minutes in 100% Xylene, RT.
- Penetration of paraffine into the plant material: add a few chips (about 20 pieces) of paraffine to 20 ml Xylene and leave overnight at 42°C.
- Remove Xylene/paraplast and add molten paraffine (60˚ C), incubate at 60˚C from now on. Refresh the molten paraffine every hour (5 times). Incubate overnight at 60˚C. Leave the tubes open to evaporate traces of Xylene.
- Refresh the molten paraffine once more and pour the boats. Add one meristem per boat.
- Store the paraffine boats at 4˚C.

C) Sectioning and treatments of the tissue

- Use commercially Poly-L-Lysine (PLL) coated slides (Thermo Scientific;J2800AMNZ 25x75x1.0) to fix the sections to the slides.
- Cut the sections (8μm) and stretch them on the coated slides in a drop of DEPC-water. Put these slides on a plate of 37˚C. Suck some water away and let it dry completely. Collect the slides in a staining dish holder and incubate it overnight at 37˚C.
- Deparaffinize the sections in Xylene (fresh), 50 times up and down in 100% Xylene, be sure that all the paraffine is gone.
- Hydrate the tissue gradually in steps from 100% Ethanol, 96% Ethanol, 70% Ethanol to DEPC-water.
- Incubate in 1x PBS (70mM Na2HPO4 en 30mM NaH2PO4 1,3M NaCL pH7.0) for 2 minutes.
- Mix 625μl of protease 50mg/ml (Sigma P5147; after dissolving predigest protease for 4 hrs at 37˚C) in 250 ml protease buffer (100 mM Tris/HCL pH8, 50 mM EDTA) pre□warmed to 37°C and incubate the slides for 20 min at 37°C. Note: The protease is required to digest fixed proteins and increase probe access to cellular RNAs. It is critical to control the time of incubation to maximize the hybridization signal without breakdown of the tissue, thus some optimization may be required for each tissue sample.
- Neutralize the protease activity in 0.2% glycine (filter sterile) in 1x PBS for 2 min.
- Rinse the slides once in 1xPBS for 2 min.
- Rinse once in DEPC-water of RT. Perform the Acetylation Reaction by incubation for 10 minutes at RT in 0.25% Acetic Anhydride in 85 mM TEA Buffer pH 8.0 (Per 250ml = 3.17g TEA (triethanol amine), 0.6ml 12M HCl, 625μl acetic anhydride).
- Rinse three times with DEPC-water, RT.
- Dehydrate gradually from 70% Ethanol to 96% to 100% Ethanol.
- Rinse once again in fresh 100% Ethanol.
- Dry it completely. This will take at least 45 minutes.

D) Synthesis of digoxigenin labeled RNA

Digoxigenin labeled RNA was synthesized using a DIG RNA labeling kit

- Clone the insert in a vector (pGEM Teasy) with T7 and SP6 promoters flanking the insert. T7 promoters are favorite, so try to get a construct with this promoter in the right orientation.
- Perform a PCR with M13 primers in 50µl. Check on gel for amount of DNA.
- Purify PCR product with a phenol/chloroform extraction and precipitate DNA with 10µg RNase-free glycogen added. Wash with 70% ethanol.
- Dissolve in DEPC treated water to a conc. of 75-100ng/µl.
- Add:
  - 2 µl PCR product(± 100-200ng)
  - 4l Transcription buffer 5x (prewarm at 37˚C)
  - 2µl DIG-RNA labeling mix 10x (Roche 11277073910)
  - 2µl DTT (100mM)
  - 2µl T7 or SP6 RNA polymerase (Promega P108B)
  - 1µl RNase inhibitor (RNasin or RnaseOUT)
  - Add water up to 20 µl.
- Incubate 2 hours at 37˚C
- Precipitate with 100% ethanol with 10µg RNase-free glycogen added. Wash with 70% ethanol (phenol extraction will reduce the yield!).
- Dissolve the large pellet in 100 µl DEPC-water and check 5µl on a formaldehyde gel together with 2µl of the hydrolyzed endproduct and blot the gel and perform the immunological detection described in G.
- The size of the probe must be reduced to 100-150 nt to allow a better penetration into the tissue. For this the RNA is chemically degraded by incubation in alkali at 60˚C. The length of the incubation time is determined by the formula below:

```
t=(Lo-Lf)/(K x Lo x Lf) in minutes; Lo=starting length (kb); Lf=final length (kb; best results were

    (2.2kb = 56min; 1kb = 51 min; 0.6kb = 45min)
```

- Add to 100 µl probe: 10µg RNase-free glycogen and 100 µl hydrolysis buffer (Hydrolysis buffer: mix 0.2 M NaHCO3 and 0.2 M Na2CO3 in a ratio 2:3; pH 10.2).
- After incubation at 60˚C add 1/10 vol 3M NaAc ph 5.5 and 10 µl 10% acetic acid and precipitate with ethanol (-20˚C).
- Wash pellet with 70% ethanol and dry pellet.
- Dissolve pellet in 20µl water and store in at -80˚C. Use 2µl for the blot described above; Use 2-4µl of the probe per 1ml hybridization mix. High probe concentrations can give high background.

E) Hybridization of the slides

- Take the DIG labeled probe from the –80˚C freezer
- Adds 2-4µl probe (300-600 ng) per ml hybridization mix.

Hybridization Mix:

• 300 – 600 ng/ml hydrolized probe (see protocol DIG –labeled RNA)
• 50% Formamide
• 300 mM NaCl
• 10mM Tris-HCl pH 7.5
• 1mM EDTA
• 1x Denhardt’s (50x Denhardt’s, Thermo Fisher 750018)
• 10mM DTT
• 250 ng/ml tRNA
• 100 µg/ml Poly(rA)

- Prewarm the mix with probe to 70˚C for one minute before applying to the slides. Use approximately 200µl per section.
- Cover the slides with a coverslip (without airbubbles) and place them upside down into a vertically placed staining dish, which is then positioned in a humidity chamber, with the slides upside down. As a humidity chamber we use a 2 liter beakerglass with two layers of paper towels soaked with 50% Formamide/2xSSC on the bottom of the beaker. Cover the beaker first with Parafilm and then with aluminium foil. Hybridize overnight at 45˚C.

F) Washing

- After hybridization put the slides with coverslips in a staining dish with 2 x SSC at RT (15 min). The coverslips should slide off the slides and fall to the bottom of the staining dish.
- Washing procedure: Use one of the below, dependent of the probe used!
  1) Mild wash: 3 x 25 min with 1 x SSC at 37˚C.
  2) Stringent wash (used for ZmRap2.7): 3 x 25 min with 0.2 x SSC at 55˚C. preheated bottle in a waterbath
- RNAse treatment: incubate 30 min at 37˚C in prewarmed NTE (500mM NaCl in 10mM Tris, 1 mM EDTA, pH 8.0) with 20µg/ml RNAse A (Roche).
- Rinse a few times with MQ-water.

G) Immunological detection:

- Incubate for 1 hour in Buffer 1, shake carefully at RT.

  • Buffer 1: 100 mM Tris-HCL pH 7.5, 150 mM NaCl, 1% Blocking (Roche 11096176001) (10% blocking is made in 100mM maleic acid and 250 mM NaCL pH7.5; autoclave; Note: blocking dissolves poorly)
- Incubate 30 min in Buffer 2, shake carefully at RT.

  • Buffer 2: 0.5% BSA, 0.3% TRITON X-100, 100 mM Tris-HCL pH 7.5, 150 mM NaCl
- Incubate with Anti-DIG Antibody in Buffer 2 (1:1000).
- Drop 200 µl on a slide and cover with a coverslip. Incubate in a humidified immuno slide staining tray for 2 hours at RT.
- Wash 3 times 20 min with Buffer 2B (100 mM Tris-HCL pH 7.5/150 mM NaCl), shake carefully at RT.
- Wash 10 min in Buffer 3, shake carefully at RT

  • Buffer 3: 100 mM Tris-HCL pH 9.5, 100 mm NaCl, 50 mM MgCl2

- Perform the enzyme incubation (Colour reaction) by adding 200µl western blot stabilized substrate alkaline phosphatase solution (Promega S3841). Cover the slides with a coverslip (without airbubbles). Incubate O/N in a humidified immuno slide staining tray, in the dark at RT. Color for multiple days in case necessary (a week incubation is possible).
